# Tracing the evolution of tissue inhibitor of metalloproteinases in Metazoa with the *Pteria penguin* genome

**DOI:** 10.1016/j.isci.2023.108579

**Published:** 2023-11-25

**Authors:** Chao-Yi Ma, Yi Chen, Xin Zhan, Yun-Wei Dong

**Affiliations:** 1 Key Laboratory of Mariculture, Ministry of Education, Fisheries College, Ocean University of China, Qingdao 266003, P.R. China; 2 Academy of the Future Ocean, Ocean University of China, Qingdao 266100, P.R. China; 3 State Key Laboratory of Marine Resources Utilization in South China Sea, Hainan University, Haikou 570228, P.R. China; 4 School of Marine Biology and Fisheries, Hainan University, Haikou 570228, P.R. China

**Keywords:** Zoology, Evolutionary biology, Phylogeny

## Abstract

Tissue inhibitors of metalloproteinase (TIMPs) play a pivotal role in regulating extracellular matrix (ECM) dynamics and have been extensively studied in vertebrates. However, understanding their evolution across invertebrate phyla is limited. Utilizing the high-quality *Pteria penguin* genome, we conducted phylogenomic orthology analyses across metazoans, revealing the emergence and distribution of the TIMP gene family. Our findings show that TIMP repertoires originated during eumetazoan radiation, experiencing independent duplication events in different clades, resulting in varied family sizes. Particularly, Pteriomorphia bivalves within Mollusca exhibited the most significant expansion and displayed the most diverse TIMP repertoires among metazoans. These expansions were attributed to multiple gene duplication events, potentially driven by the demands for functional diversification related to multiple adaptive traits, contributing to the adaptation of Pteriomorphia bivalves as stationary filter feeders. In this context, Pteriomorphia bivalves offer a promising model for studying invertebrate TIMP evolution.

## Highlights

• The TIMP gene family originated in the common ancestor of eumetazoans
• TIMP gene family sizes vary dramatically across metazoan clades
• Pteriomorphia bivalves exhibit the largest and most diversified TIMP repertoires

## Graphical abstract

**Figure:**
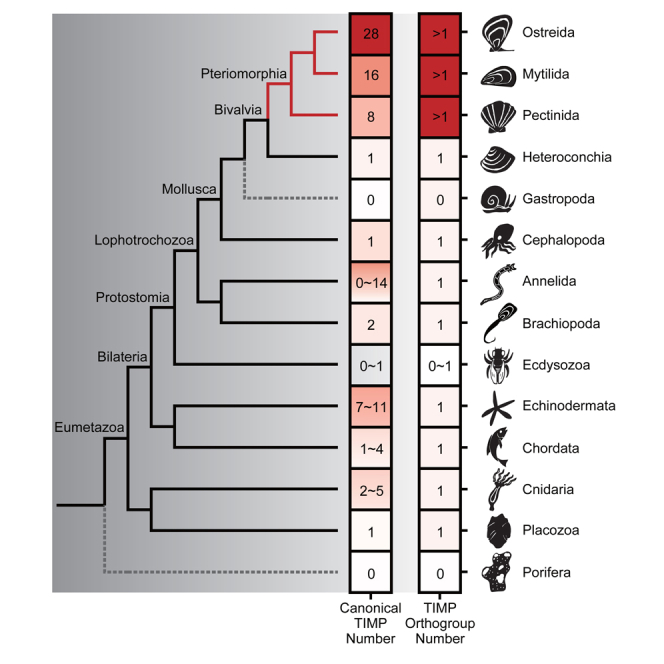

## Introduction

The evolutionary dynamics of key gene families have long been a topic of great interest in evolutionary biology.^1^^,^^2^ This includes studying the variations in gene family size and functional divergence during the evolutionary process. Analyzing the patterns of these events, while often difficult to achieve, can provide insights into the forces driving gene family evolution and their impact on the adaptation and evolutionary success of certain species.^3^^,^^4^

The tissue inhibitor of metalloproteinase (TIMP) family is an ancient gene family that is widely distributed in the Metazoa kingdom.^5^^,^^6^ TIMPs primarily function as endogenous inhibitors of metalloproteinases, particularly matrix metalloproteinases, which are enzymes responsible for extracellular matrix (ECM) degradation.^7^ This inhibitory function is mainly mediated by the netrin-like (NTR) domain, which is conserved and corresponds to the N-terminal domain in TIMPs. The NTR domain can independently interact with the active site of MMPs, forming a tight 1:1 complex and inhibiting MMP activity.^8^^,^^9^ By regulating MMP activity, TIMPs play a crucial role in maintaining the balance between ECM synthesis and degradation, ensuring proper tissue remodeling and repair processes.^10^^,^^11^ As a key gene family instrumental in ECM modification, investigating the evolutionary trajectory of TIMPs across metazoans can provide valuable insights into the mechanisms that govern ECM dynamics in organismal development and survival.

Promising research has been conducted on the evolution and functional diversification of the TIMP gene family within specific clades in vertebrates.^6^^,^^10^ A comprehensive study revealed four TIMP members, commonly found in jawed vertebrates, originated from a common ancestor through three successive duplications during early vertebrate radiation.^12^ Throughout their evolutionary divergence, different members of the vertebrate TIMP family have exhibited varying rates of evolutionary change and have undergone gain and loss of functions.^12^ For instance, some members have lost inhibitory activity toward specific metalloproteinases while developing new interactions with specific targets such as integrins and receptors, while retaining their roles in regulating ECM structure.^6^^,^^13^ These findings highlight the complex evolutionary history of the TIMP gene family.

The TIMPs in invertebrates share a similar domain architecture but exhibit substantial sequence divergence compared to vertebrate TIMPs, indicating distinct evolutionary paths.^6^^,^^10^ A notable example is the study conducted on the evolutionary trajectory of TIMPs in echinoderms,^14^ which belong to the Deuterostomia clade along with vertebrates. Phylogenetic analysis has revealed that the TIMP gene family underwent diversification in the ancestral deuterostome. Unlike chordates, which retained only a single copy in the ancestral stage and subsequently diversified into four members in vertebrates, echinoderms retained numerous early-lineage TIMPs and continued to diversify after their divergence from chordates. Despite significant sequence differences compared to vertebrates, invertebrate TIMPs retain inhibitory activities against metalloproteinases.^10^^,^^12^ TIMPs from cnidarians exhibit a canonical TIMP fold structure, like mammalian TIMPs, with only minor structural changes, suggesting the evolutionary conservation of a common structure across metazoans.^15^ However, comprehensive studies focusing on the evolutionary events of invertebrate TIMPs, particularly in protostomes, are still limited despite the vast diversity within this group. A more comprehensive understanding of TIMP repertoires in invertebrates, which are currently underrepresented in evolutionary studies centered around the TIMP gene family, is essential for gaining insights into the evolution of the TIMP gene family across the entire animal kingdom and facilitating further functional studies within an appropriate evolutionary context.

Despite Mollusca being the second largest phylum of invertebrates, our understanding of the evolution of TIMP genes in this clade is still lacking. Present studies in molluscs have primarily centered around the bivalves from the Pteriomorphia clade, including *Tegillarca granosa* (Arcida),^16^ *Mytilus* spp. (Mytilida),^17^^,^^18^ *Magallana* spp. and *Pinctada* spp. (Ostreida),^19^^,^^20^^,^^21^^,^^22^^,^^23^^,^^24^^,^^25^^,^^26^^,^^27^ and *Chlamys farreri* (Pectinida).^28^^,^^29^^,^^30^^,^^31^ These studies have revealed that TIMPs found in Pteriomorphia bivalves play important roles in the immune system^16^^,^^19^ and formation of the bivalved shell and the hinge ligament,^22^^,^^23^^,^^25^ as well as the development of the bivalve byssus.^18^^,^^25^^,^^26^^,^^31^ These findings suggest that the TIMP gene family is crucial for the survival and adaptation of Pteriomorphia bivalves and may have undergone expansion in family size during their evolution. Moreover, Pteriomorphia bivalves can serve as valuable model organisms for studying the evolutionary patterns and diversification of the TIMP gene family in invertebrates. In addition to the Pteriomorphia bivalves, *Pteria penguin*, commonly known as the penguin’s wing oyster, belongs to the family Pteriidae, within the order Ostreida. Native to the western and central Indo-Pacific region, this species is widely cultivated for pearl production.^32^ While genomic resources and TIMP information for *P*. *penguin* are currently unavailable, it is reasonable to speculate that this species may possess a rich repertoire of TIMPs based on its phylogenetic relationship with other bivalves, as well as notable features such as a well-developed nacre layer and byssus in the adult stage. Conducting a comprehensive phylogenomic analysis with a specific focus on molluscs would significantly contribute to our understanding of the underappreciated TIMP resources in Pteriomorphia bivalves and provide insights into the broader distribution of TIMPs across metazoans.

In this study, we aim to address the gaps in our understanding of the evolution of the TIMP gene family in Mollusca and investigate the origin and diversification of canonical TIMPs across the phylogeny of Metazoa. While the size of the TIMP gene family in various molluscan clades remains inconclusive, previous studies have provided insights into the diverse functions of TIMPs in Pteriomorphia bivalves.^19^^,^^22^^,^^28^ Hence, we hypothesize that the TIMP gene family undergoes multiple duplication events and exhibits an extensive repertoire in Pteriomorphia bivalves. To address this hypothesis, we initiated the study by generating a high-quality genome assembly for P. penguin and conducted comprehensive phylogenomic and orthology inference analyses incorporating other metazoan genomes. Our findings offer a comprehensive overview of the distribution patterns of the TIMP gene family across the animal kingdom, suggesting the TIMP repertoires in metazoans originated from ancestral genes that emerged during the early radiation of eumetazoans. The most significant revelation from our research is that Ostreida bivalves belonging to the Pteriomorphia infraclass possess the most extensive and diverse repertoire of TIMPs in the animal kingdom. We propose that the abundance of TIMP repertoires in these species results from multiple independent duplication events driven by the various functional demands of different ECM protein-related tissues. These insights of this study could pave the way for further research into the role of TIMPs in a wide range of biological processes across metazoans by providing a crucial evolutionary context.

## Results

### A high-quality *P*. *penguin* genome

**Figure 1:**
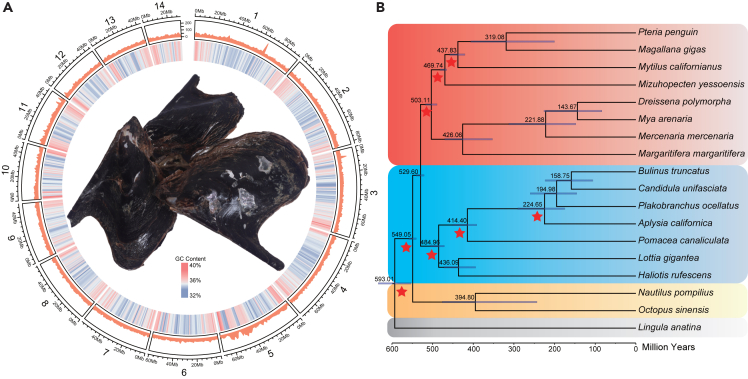
The penguin’s wing oyster *Pteria penguin* and its phylogenetic positions (A) The photograph of *P*. *penguin*. Circos plot showing the 14 pseudomolecules. The outer ring (red peaks) indicates gene density in each pseudo chromosome, and the inner ring shows GC content; the sliding window size is 1 MB, megabases. (B) Time-calibrated phylogenomic tree showing the position of the *P*. *penguin* among seventeen molluscan species and one brachiopod species *Lingula anatina*. The color labeling scheme of taxa: Bivalvia: red; Gastropoda: blue; Cephalopoda: orange; other two Lophotrochozoan species: gray. The blue horizontal bars indicate the 95% confidence intervals of the divergence times. Red stars indicate the calibrated nodes based on fossil records and geographic events. The node labels are marked on the tree nodes.

Genomic DNA was extracted from a single individual of *P*. *penguin* (Figure 1A) and sequenced using Illumina short-read and PacBio long-read sequencing platforms (Table S1). The *de novo* assembly process, followed by heterozygosity removal and polishing, resulted in a final genome assembly of 838.71 Mb. The assembly had a contig N50 of 5.03 Mb and a scaffold N50 of 60.66 Mb, comparable to the estimated size by k-mer analysis (812.32 Mb) (Figure S1). Additionally, leveraging 46 Gb Hi-C sequencing data, approximately 99.83% of the obtained contigs were successfully anchored into 14 pseudo chromosome linkage groups (Table S2). To assess the completeness of the genome assembly, Benchmarking Universal Single-copy Ortholog (BUSCO) analysis was performed, revealing 95.8% metazoan BUSCOs present (Table S3). These results confirm the high quality and completeness of the assembled *P*. *penguin* genome.

For genome annotation, repeat elements constituted 43.89% (368.14 Mb) of the assembled genome (Table S4). A total of 36,733 protein-coding genes were identified within the *P*. *penguin* genome using *ab initio* methods and transcripts assembly constructed from Illumina and PacBio sequencing data (Table S5). Among these genes, 99.85% (36,677) were anchored to the 14 pseudo chromosome linkage groups. BUSCO analysis further demonstrated the completeness of the protein-coding gene set in the *P*. *penguin* assembly, with a metazoan BUSCO score of 97.8%. Additionally, a total of 16,884 noncoding RNA genes were annotated across the *P*. *penguin* genome (Table S6).

To investigate the phylogenetic relationship of *P*. *penguin* within the molluscs, a set of 273 single-copy genes was identified among seventeen molluscan species and one brachiopod *Lingula anatina* (for detailed information, see Table S7). These genes were used to construct a phylogenomic tree, which placed *P*. *penguin* together with the Pacific oyster *Magallana gigas* in the Ostreida order (Figure 1B). Both species, along with the mussel *Mytilus californianus* and the scallop *Mizuhopecten yessoensis*, clustered within the Pteriomorphia infraclass. This infraclass represents the sister clade to Heteroconchia, which includes the freshwater mussel *Dreissena polymorpha*, the soft-shell clam *Mya arenaria*, the hard clam *Mercenaria mercenaria*, and the freshwater pearl mussel *Margaritifera margaritifera*.

### Phylogenetic orthology analysis reveals the distribution of the TIMP gene family across Mollusca

**Figure 2:**
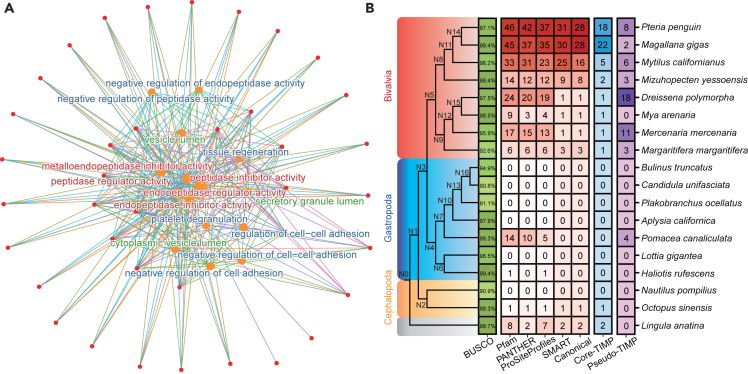
The TIMP family has dramatic expansion in *P*. *penguin* and other relative bivalves (A) The GO pathways related to the TIMP gene family and TIMP activity, which were clustered from the expanded gene families within the order Ostreida. The molecular function (MF) GO pathways are shown in red, the biological process (BP) GO pathways are shown in blue, and the cellular component pathways are shown in green. (B) The number of putative TIMP copies in each species and node labels were marked on the node of the phylogeny tree. With deeper color denoting the higher values, the BUSCO numbers indicating the completeness of protein sets are marked in green boxes, the number of sequences having matches in different databases as well as the finally identified canonical TIMPs are marked in red boxes, TIMP numbers in core Mollusca-Brachiopoda TIMP HOG (*HOG0001471*) are marked in blue boxes, and the numbers of pseudo TIMPs are marked in purple boxes. The cladogram tree is inherited from the results of this research.

Using Orthofinder software, a total of 38,849 orthogroups (OGs) were identified among eighteen species, representing 88.7% of all assigned genes. For each OG, a rooted gene tree was constructed by introducing the outgroup species *L*. *anatina*. Subsequently, 40,746 orthogroups at each hierarchical level (HOGs) were inferred from the OGs by analyzing the rooted gene trees. The gene copy numbers in each HOG were counted to estimate gene family expansion and contraction events across the molluscan phylogenomic tree. Among the 17,411 HOGs owned by the Ostreida clade (*P*. *penguin* or *M*. *gigas*), 82 gene families experienced significant expansion, while 13 gene families showed significant contraction compared to their parent clade (p value <0.05). Gene Ontology (GO) enrichment analysis of the aforementioned expanded HOGs in *P*. *penguin* genome revealed 276 highly significantly enriched GO categories (adjusted p value <0.01; listed in Table S8), of which fifteen were related to the TIMP gene family and TIMP activity (Figure 2A), indicating a massive expansion of the TIMP family in the species belong to the Ostreida order.

To quantify the copy numbers of TIMP genes in the eighteen species, their genomes were scanned, resulting in the identification of 144 putative TIMP sequences. These putative TIMPs had protein signature matches with “Tissue inhibitor of metalloproteinase” in the Pfam database, “METALLOPROTEASE INHIBITOR” in the PANTHER database, and “NTR domain profile” in the PROSITE database, all simultaneously. These 114 TIMP sequences were further classified into two groups based on whether they matched the conserved NTR domain in the SMART database. One group included 89 sequences, which were named “canonical TIMPs” because they had matches to SMART NTR domain. The other group consisted of 55 sequences, which were referred to as “pseudo TIMPs” since they did not have matches to the domain. Analysis of the canonical TIMP gene distribution revealed significant divergence in copy numbers among molluscan species. Cephalopods, gastropods, and some bivalves from the infraclass Heteroconchia showed either one copy or no canonical TIMP gene, except for *M*. *margaritifera*, which possessed three copies. In contrast, Pteriomorphia bivalves exhibited a dramatic expansion of canonical TIMP copies, ranging from eight in *M*. *yessoensis* to twenty-eight in *M*. *gigas* and *P*. *penguin* (Figure 2B); notably, species from the order Ostreida possessed the highest number of canonical TIMP genes in their genomes. The distribution pattern of pseudo TIMPs differed from canonical TIMPs and was mainly found in seven bivalves and one gastropod (*Pomacea canaliculata*). Notably, two Heteroconchia bivalves, *D*. *polymorpha* and *M*. *mercenaria*, possessed the highest number of pseudo TIMPs compared to other species.

The HOGs containing putative TIMP sequences were further analyzed. One HOG named *HOG0001471*, containing the majority of canonical TIMPs (53 out of 89 sequences) from ten out of eighteen surveyed species that encompassed canonical TIMP genes, was considered as the “core Mollusca-Brachiopoda TIMP HOG” that possessed “core Mollusca-Brachiopoda TIMPs.” Additionally, eighteen HOGs containing other canonical TIMPs were considered “branch TIMP HOGs” with “branch TIMPs”, while ten HOGs containing no canonical TIMPs but pseudo TIMPs were named “pseudo TIMP HOGs” (Table S9). Through inferring the gene duplication events in each HOG, the core Mollusca-Brachiopoda TIMP HOG was found to have experienced multiple duplication events across different species (Figure S2), representing the mainstream evolutionary trajectory of TIMPs from their potential common ancestor. Two major duplication events were identified in the evolution of core Mollusca-Brachiopoda TIMPs within the molluscan phylogeny (marked as “Non-Terminal” in Table S10). These events were shared by more than one species and linked to the speciation events. The first event occurred during the separation of cephalopods from gastropods and bivalves. At the same time, the second event happened within the Pteriomorphia clade, specifically during the divergence of the Pectinida order from Mytilida and Ostreida. In contrast to the core Mollusca-Brachiopoda TIMP HOG, the branch TIMP HOG underwent species-specific or lineage-specific duplication events, giving rise to several TIMP subgroups specific to particular species. Similar to the previous two groups, the pseudo TIMP HOGs were derived from multiple duplications. Leveraging our chromosome-level *P*. *penguin* assembly as well as the publicly available *M*. *gigas* genome, the genomic location of putative TIMPs in these two species was retrieved, and the tandem duplications contributing to the TIMP expansion were detected and shown (Figure S3).

Phylogenetic trees were constructed using the aforementioned 144 putative TIMPs and 53 core Mollusca-Brachiopoda TIMPs, with two *L*. *anatina* sequences chosen as outgroups (Figures S4 and S5). Both trees divided molluscan sequences into two groups: a minor group consisting of single-copy canonical TIMPs from the four Heteroconchia bivalves and a major group comprising other sequences, primarily from Pteriomorphia bivalves. In the tree constructed from putative TIMPs, the pseudo or branch canonical TIMPs of *D*. *polymorpha* (eighteen pseudo TIMPs), *M*. *mercenaria* (eleven pseudo TIMPs), and *M*. *margaritifera* (three pseudo TIMPs and two branch TIMPs) were not clustered with the canonical TIMPs of these species but instead grouped within the major group. In the tree constructed from core Mollusca-Brachiopoda TIMPs, the TIMP sequence from the octopus *Octopus sinensis* clustered with the Pteriomorphia TIMPs rather than serving as an outgroup for both Heteroconchia and Pteriomorphia. Ten sequence motifs were discovered in the 144 putative TIMPs and used to compare the sequence composition of canonical and pseudo TIMPs from different phylogeny clades (Figure S4). Five motifs (*motif1* to *motif5*) were present across the majority of putative TIMPs, although absent in several sequences. Compared to other molluscan putative TIMPs, *motif7* was found only in some pseudo TIMPs of *D*. *polymorpha*, and *motif8* was barely present in canonical TIMPs from the two Ostreida bivalves, *P*. *penguin* and *M*. *gigas*, as well as their most closely related species, *M*. *californianus*. The motifs *motif9* and *motif10* were completely absent in the four sequences from *P*. *canaliculata*, and *motif10* was also absent in all sequences from Heteroconchia. Additionally, the arrangement order of motifs in core Mollusca-Brachiopoda TIMPs showed no reshuffling of order, but the position of *motif10* in molluscan TIMPs differed from that in *L*. *anatina* (Figure S5). The distribution and position of signal peptides and NTR domain in core Mollusca-Brachiopoda TIMPs were also demonstrated (Figure S5). Finally, the exon numbers in each putative TIMP gene were examined and found to range from four to six exons, with 132 out of 142 molluscan sequences encompassing five exons, similar to the exon number found in vertebrate TIMP genes.^12^ This suggests that five exons are common in molluscs.

### Phylogenetic orthology analysis reveals the distribution of the TIMP gene family across Metazoa

The Orthofinder software was employed to identify OGs among forty-one metazoans (Table S11). A total of 59,702 OGs were detected, encompassing 93.3% of all assigned genes. For each OG, a rooted gene tree was constructed using the sponge *Amphimedon queenslandica* as the outgroup. Subsequently, a total of 61,700 phylogenetic HOGs were inferred from the OGs by analyzing the rooted gene trees.

**Figure 3:**
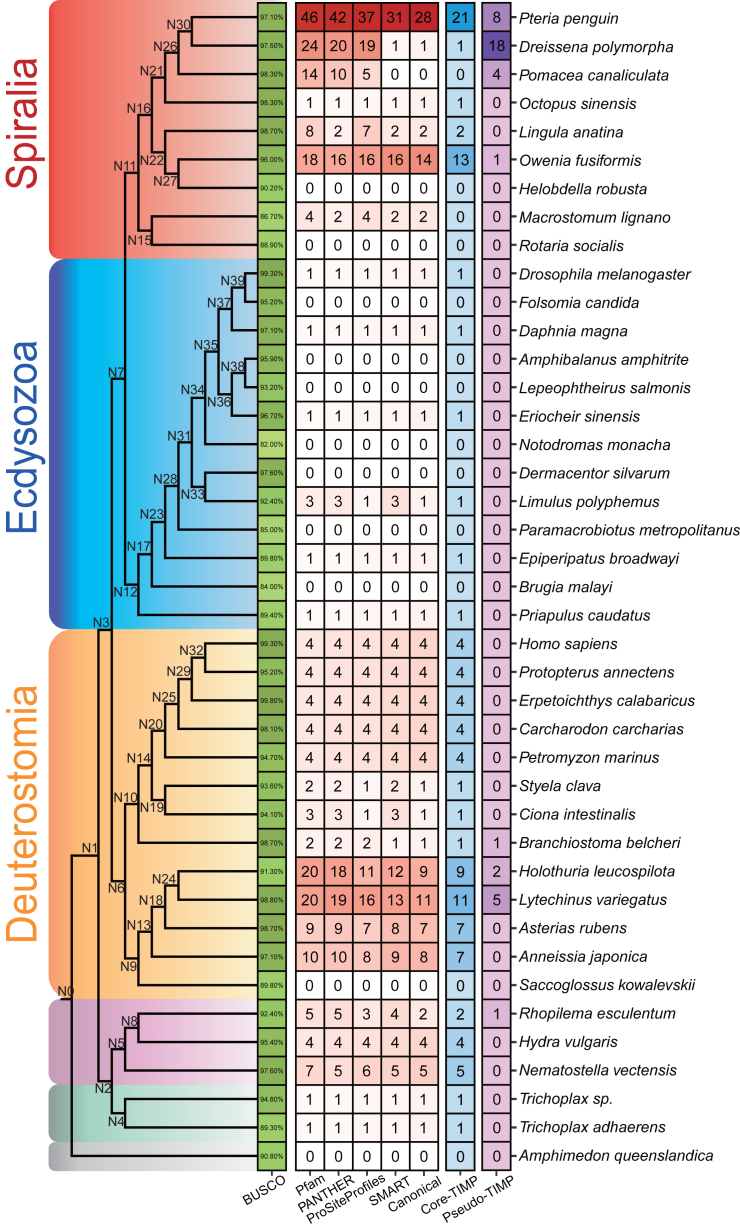
The distribution and phylogenetic relationship of the TIMP family from 41 metazoans The distribution of TIMP copy numbers in each species and node labels were marked on the node of the phylogeny tree. The detailed interpretation can be referred to Figure 2B; with some differences are the core Eumetazoa TIMP HOG named HOG0001662, and the cladogram tree is constructed by referring to existing studies.^33^^,^^34^^,^^35^^,^^36^^,^^37^^,^^38^^,^^39^^,^^40^

Through the examination of a total of forty-one metazoan genomes, we identified 165 putative TIMPs, including 125 canonical TIMPs and 40 pseudo TIMPs (Table S12). The distribution of canonical TIMPs in the surveyed species was consistent with previous findings, showing the widespread presence of orthologs across Metazoa.^10^ However, the copy numbers varied among different metazoan clades (Figure 3). In the poriferan (*Amphimedon queenslandica*), TIMP gene was absent, while in the clade containing placozoans (*Trichoplax adhaerens* and *T*. *sp*.) and cnidarians, the copy numbers ranged from one to multiple copies (Cnidaria, ranging from two to five). In Ambulacraria within Deuterostomia, Echinodermata species exhibited multiple TIMPs in their genomes, ranging from seven (*Anneissia japonica*, *Asterias rubens*) to eleven (*Lytechinus variegatus*). In Chordata of Deuterostomia clade, basal species such as cephalochordates (*Branchiostoma belcheri*) and tunicates (*Styela clava*, *Ciona intestinalis*) possessed one canonical TIMP, while the vertebrates included in this study, along with the jawless fish *Petromyzon marinus*, were found to have four canonical TIMPs. In Ecdysozoa of Protostome, only one or no copy of canonical TIMPs was detected. In Spiralia of Protostome, canonical TIMP copy numbers varied significantly. For instance, in Platyzoa, *Macrostomum lignano* had two copies, while *Rotaria socialis* had none. Among Trochozoa, which includes Annelida, Brachiopoda, and Mollusca, the TIMP copy numbers varied not only between sister clades but also within a single clade. Annelida exhibited a range from no copies in the leech *Helobdella robusta* to up to fourteen copies in the spindle worm *Owenia fusiformis*. And, as previously mentioned, the range of TIMP repertoire sizes in molluscs varied from zero copy to a remarkable twenty-eight copies, which were found in *P*. *penguin*, making it the largest repertoire of TIMP copies among all animals employed in this study. The distribution of pseudo TIMPs was also investigated and found to be present in species from Cnidaria (*Rhopilema esculentum*), Echinodermata (*Holothuria leucospilota*, *L*. *variegatus*), Chordate (*B*. *belcheri*), Annelida (*O*. *fusiformis*), and Mollusca (*D*. *polymorpha*, *P*. *canaliculata*, and *P*. *penguin*). Notably, the bivalves *D*. *polymorpha* had the highest number of pseudo TIMPs compared to other species.

Among the HOGs derived from the forty-one surveyed metazoans, the “core Eumetazoa TIMP HOG”, named *HOG0001662*, comprised the majority of canonical TIMPs (114 out of 125 sequences) from twenty-nine out of forty-one surveyed animals that encompassed canonical TIMP genes and all belong to Eumetazoa clade, the sister group of the Porifera (sponges). The number of core Eumetazoa TIMPs from each species followed the pattern observed in canonical TIMPs, with *P*. *penguin* possessing the highest number. Additionally, eight branch and pseudo TIMP HOGs were identified. Multiple gene duplication events were detected within each HOG containing putative TIMPs (Figure S6). Two major duplication events were identified in the evolution of core Eumetazoa TIMPs within the metazoan phylogeny; one occurred during the divergence of bilaterians from cnidarians and placozoans, while the other took place during the separation of protostomes from deuterostomes (Table S13).

Phylogenetic trees were constructed using the aforementioned 165 putative TIMPs and 114 core Eumetazoa TIMPs, with the two sequences of Placozoa species chosen as the outgroups (Figures S7 and S8). Among the ten sequence motifs discovered in the 165 putative TIMPs, *motif1* and *motif3* were present in the majority of putative TIMPs, with only a few sequences lacking them (Figure S7). The *motif2* and *motif4* were commonly found in canonical TIMPs, but one or both were absent in the pseudo TIMPs. Consistent with the previous findings, *motif10* was exclusively observed in some of the pseudo TIMPs from *D*. *polymorpha*. Four motifs (*motif1* to *motif4*) and seven motifs (*motif1* to *motif5*, *motif7*, *motif8*) were concurrently present in certain canonical TIMPs from eleven phyla representing the four major metazoan clades. These clades included Placozoa and Cnidaria (which were sister groups), Echinodermata, Chordata (both belonging to Deuterostomia), Arthropoda, Onychophora, Priapulida (belonging to Ecdysozoa of Protostomia), Annelida, Brachiopoda, Mollusca, and Platyhelminthes (belonging to Spiralia of Protostomia). No reshuffling of motif order was observed in the 114 core Eumetazoa TIMPs (Figure S8). The distribution and position of signal peptides and NTR domain in core Eumetazoa TIMPs were also demonstrated (Figure S8). Lastly, the exon number in each gene of the canonical and pseudo TIMPs was calculated. Out of the 165 sequences, 127 encompassed four to six exons, with 60 sequences having five exons. In Echinodermata, 25 out of 41 sequences had four exons, while in Placozoa and Cnidaria 10 out of 13 sequences encompassed two exons, indicating the common exon numbers in these two clades.

## Discussion

**Figure 4:**
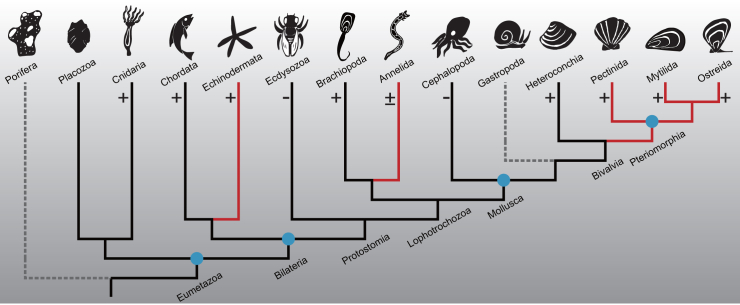
Schematic of features in the evolution of TIMP gene family in Metazoa kingdom The phylogeny tree represents relationships among major metazoan clades employed in this study. Branch lengths are not indicative for evolutionary distances. Solid branches signify clades retaining TIMP gene copies, while dashed branches denote complete TIMP gene loss within a clade. Red branches highlight three remarkable TIMP expansions across metazoans: in echinoderms, the Polychaeta annelid *O*. *fusiformis*, and Pteriomorphia bivalves. Circles on tree nodes indicate four major duplication events of TIMP family along metazoan phylogeny, including the divergence of bilaterians from cnidarians and placozoans, protostomes from deuterostomes, bivalves and gastropods from cephalopods, and the separation of Mytilida and Ostreida order from Pectinida order. Plus signs (**+**) along the branches indicate lineage- or species-specific duplication events of TIMP genes within these clades, while minus signs (**−**) indicate complete TIMP gene loss in at least one species.

The primary objective of this study is to investigate the evolutionary dynamics and distribution of the TIMP gene family within the animal kingdom, with a particular focus on molluscs. To achieve this, we performed comprehensive analyses of the TIMP gene family across the metazoan phylogeny. By assembling a high-quality genome assembly of *P*. *penguin* and comparing it with other animals, we identified TIMP orthologs and examined their distribution in various animal clades. While the canonical TIMP gene is absent in the genome of the poriferan *A*. *queenslandica*, its orthologs are widely distributed among the eumetazoans, spanning from simple placozoans to bilaterians with complex tissue structures. It is worth mentioning that the phylogeny orthology analyses reveal a shared TIMP orthology group among the analyzed animals with canonical TIMPs, indicating a common ancestral origin in the evolution of the TIMP gene family. These findings also suggest the TIMP gene family likely emerged in the common ancestral lineage of eumetazoans after diverging from poriferans but before the split between the Placozoa + Cnidaria clade and Bilateria. Within the TIMP repertoires of various animal clades, a remarkable expansion was observed in the bivalves of the infraclass Pteriomorphia. From the common ancestor of eumetazoans to Pteriomorphia bivalves, four major duplication events of TIMP family during speciation events have occurred, along with multiple lineage- or species-specific duplication events after speciation events, potentially driven by adaptive demands. Accompanying the TIMP duplication events are the secondary loss events of the TIMP members in some lineage or species along the metazoan phylogeny. The duplication and loss events shape the distribution pattern of TIMPs in extant animals (Figure 4).

Our study provides comprehensive genomic analyses of TIMP gene copy number distribution across the Metazoa kingdom. Regarding the protostomes, ecdysozoans either completely lose TIMPs or retain a single-copy TIMP within their genomes. In contrast, the Spiralia displays two distinct expansions. One occurs in Annelida, where we identified fourteen canonical TIMPs in *O*. *fusiformis* from the Polychaeta class. Conversely, no TIMP gene was found in *H*. *robusta* from the Clitellata class. The variation in the size of the TIMP gene family within Annelida necessitates further elucidation through future studies incorporating additional genomic resources. Another noteworthy expansion was observed in the Mollusca phylum, which exhibited considerable variation in the size of the TIMP gene family across its different clades. Some cephalopods have one TIMP gene, while gastropods have lost the canonical TIMP family during evolution. This observation highlights the need for further studies to investigate the evolutionary mechanisms behind the loss of TIMP members in gastropods. Compared with other molluscs, the Pteriomorphia bivalves exhibit a general expansion of the TIMP gene family. Within this clade, the copy number of the TIMP family has a linear relationship along the evolution phylogeny and reaches its peak in the Ostreidae bivalves with the most recent origins.^41^ The Ostreidae bivalves employed in this study, *P*. *penguin* and *M*. *gigas*, possess the highest number of canonical TIMP genes within their genomes, surpassing all other animals included in our research. The distribution of TIMP genes in the genomes of *P*. *penguin* and *M*. *gigas* provides compelling evidence supporting frequent gene tandem duplication as the primary mechanism underlying the notable expansion of the TIMP gene family. This mechanism is also implicated in the expansion of other gene families to unparalleled sizes in bivalves.^42^^,^^43^ Shifting the focus to the non-protostomes, our findings generally align with previous reports on TIMP copy numbers in cnidarians,^15^ echinoderms,^14^ and vertebrates;^12^ with a few exceptions. In the jawless fish *Petromyzon marinus*, we observed four TIMP genes, which deviates from the previously reported number of three.^12^ The four TIMPs in jawed vertebrates most likely originated from three successive duplications before the split of Chondrichthyes (cartilaginous fishes) and Euteleostomes (ray-fins and lobe-fins). In contrast, similar duplications are considered to occur only once or twice in Agnatha (jawless fishes). These suggest an additional Agnatha-specific duplication for the TIMP gene after their separation from the vertebrate stem. Another exception pertains to echinoderms, where the size of the TIMP repertoire identified in our study is relatively smaller than that previously reported.^14^ This discrepancy may arise from alternative splicing events, wherein a single TIMP gene can generate different transcript isoforms.^44^^,^^45^ Previous transcriptome-based methods may have consequently overestimated the distribution of TIMPs in echinoderms to some extent. In brief, our phylogenomic analysis offers a comprehensive investigation of the size variations in the TIMP gene family across the Metazoa kingdom. It highlights an unparalleled expansion of the TIMP gene family in the Pteriomorphia bivalves, emphasizing a unique evolutionary process.

High diversity is also a prominent feature of the TIMP repertoires of Pteriomorphia bivalves. In contrast to the echinoderms and *O*. *fusiformis*, which also exhibit an expansion of the TIMP gene family in the genome, all their TIMP orthologs are grouped within the Eumetazoa orthology group shared by other species containing TIMP genes, namely the core Eumetazoa HOG identified in our results. This indicates the limited diversity of the TIMP family in echinoderms and *O*. *fusiformis*, suggesting a relatively recent origin from a single expansion event.^42^ The canonical TIMPs from *P*. *penguin* display a high diversity and are clustered into five different orthology groups, including the core Eumetazoa orthology group and the other four restricted to *P*. *penguin*. Indeed, 25% of the canonical TIMPs (seven sequences) from *P*. *penguin* were not included in the core Eumetazoa orthology group but dispersed in other branch orthology groups, suggesting the evolution novelty of these sequences.^42^ In our molluscan orthology analysis, the diversity of the TIMP gene family in Pteriomorphia bivalves is particularly pronounced. Along with the core Mollusca-Brachiopoda orthology group, the canonical TIMPs of Pteriomorphia bivalves disperse into twelve different orthology groups. Seven of these groups are shared among different species, while the remaining five orthologs are specific to a single species. This suggests that the large-scale TIMP expansion in Pteriomorphia bivalve results from multiple, independent duplication events occurring at distinct stages along the evolutionary phylogeny. The initial expansion likely occurred in the latest common ancestor of Pteriomorphia bivalves, contributing to their larger repertoire compared with their sister clade, the Heteroconchia. Subsequent independent expansions occurred during the radiation of Pteriomorphia bivalves, with some leading to recent species-specific evolutionary innovations, resulting in observed phylogenetic TIMP orthology branches exclusive to Pteriomorphia bivalves.

The remarkable expansion and diversity of the TIMP family in Pteriomorphia bivalves can be attributed to their adaptation to habitats. This adaptation-driven expansion of the TIMP family has been indicated in echinoderms, as these animals have high demands for tissue remodeling as adaptive mechanisms.^14^ For Pteriomorphia bivalves, these marine bivalves primarily inhabit intertidal zones or shallow waters as stationary filter feeders, which expose them to constant threats, including aquatic pathogens and pervasive predators, as well as the fluctuating physicochemical conditions of the surroundings.^46^ Faced with these multiple stresses, Pteriomorphia bivalves require robust adaptive mechanisms, which can be facilitated by the TIMP family and may further drive the subsequent duplication and neofunctionalization events of this family in Pteriomorphia bivalves. Firstly, these bivalves have developed an effective innate immunity system involving multiple adaptive evolutionary processes.^47^ A crucial aspect of this adaptation is the expansion of specific immune-related gene families, such as the Toll-like receptor (TLR) and inhibitors of apoptosis (IAPs) families.^42^^,^^43^ These genetic changes are closely associated with the immune needs of bivalves in response to the stress from their living environments. In Pteriomorphia bivalves, certain TIMP members specifically expressed in hemocytes have been confirmed to play a pivotal role in immune mechanisms, including wound healing and the defense against microbial pathogens.^16^^,^^19^^,^^21^ The demand for immune processes via TIMP members may drive the adaptive duplication of a subset of TIMP repertoires related to the immune system. Secondly, the survival of Pteriomorphia bivalves relies on their bivalved shells, mainly serving as a defensive barrier against external predators. In the case of true oysters like those in the Ostreidae family, the shell firmly cements these oysters to substrates. The components within the shell of Pteriomorphia bivalves contain various matrix proteins contributing to shell formation and growth.^48^^,^^49^ Among these shell matrix proteins of Pteriomorphia bivalves, the TIMP family has consistently been identified as one component, further substantiating its role in facilitating shell formation and growth. Some TIMP members in the Iwagaki oyster *M*. *nippona* are specifically expressed in the mantle,^27^ the tissue responsible for shell development. Moreover, studies conducted through RNA interference experiments in the pearl oyster *Pinctada fucata* have provided evidence of TIMP proteins participating in the formation of shell and ligament structure.^23^^,^^25^ Additionally, some TIMP proteins or TIMP-related domains have been identified as integral components of the shell structure,^17^^,^^22^^,^^24^^,^^27^^,^^49^ possibly serving to protect the shell protein components from degradation by external proteinases. These studies indicate the involvement of the TIMP family in shell development can be a key driver for its general expansion and diversity in Pteriomorphia bivalves. This expansion aligns with the observed duplication events in other genes encoding shell matrix proteins, regarded as fundamental mechanisms in the evolution of these families.^27^^,^^50^ Thirdly, byssus, an adhesion structure made of protein filaments and secreted by the foot, serves as an adaptive trait in Pteriomorphia bivalves. Byssus is crucial for the larvae’s settlement and metamorphosis. In adults, many Pteriomorphia bivalves, such as mussels in the Mytiloidea family and pearl oysters in the Pteriidae family, retain byssus for attachment to hard substrates.^51^ Specific TIMP members in Pteriomorphia bivalves not only play roles in byssus fabrication, as indicted by multiple studies based on foot transcriptomes,^18^^,^^28^^,^^29^^,^^31^ but also have been shown to be integrated into the protein components of byssus.^18^^,^^26^^,^^28^ Some of these structural TIMPs even have evolved beyond their inhibitory function, transforming into cross-linkers that may facilitate the self-assembly of byssus.^30^^,^^31^ These byssus-related TIMPs undergo duplication and subsequent neofunctionalization events as adaptive mechanisms in Pteriomorphia bivalves to meet their dependence on byssus. Lastly, some other factors contributing to the expansion of the TIMP family in Pteriomorphia bivalves may be attributed to neutral or non-adaptive processes, such as neutral gene duplication events,^52^^,^^53^ and contribute to the initial duplication event in the ancestor of Pteriomorphia bivalves. Nevertheless, the adaptive force is still recognized as the main driving force behind the multiple duplication events of this family, as supported by the close relationship between the functional diversity exhibited by multiple TIMP members and the adaptive traits in Pteriomorphia bivalves. In summary, the expansion of the TIMP family can be driven by the need for multiple adaptive traits in Pteriomorphia bivalves and plays a crucial role in the survival and adaptation of these bivalves in living environments. Further investigation is needed to explore more precise reasons behind the expansion of this family.

The emergence of similar adaptive traits through distinct genetic mechanisms is a common phenomenon in nature.^54^^,^^55^^,^^56^^,^^57^ This process contributes to a better understanding of the lineage-specific expansion of the TIMP family in Pteriomorphia bivalves. Beyond Pteriomorphia bivalves, this study also investigated the distribution of TIMP families in other animals such as stationary filter-feeding animals in aquatic environments, but none of them exhibited the remarkable TIMP expansion seen in Pteriomorphia bivalves. Among these animals, Heteroconchia bivalves, which are the sister group to Pteriomorphia bivalves and share similar adaptive traits,^58^ including effective innate immunity, bivalved shells, and adhesive byssus, have much smaller TIMP repertoires in comparison to Pteriomorphia bivalves. Considering the more than 500 million years of divergence between these two groups, despite the deep conservation in fundamental biological processes, specific divergences remain in the genetic mechanisms underlying some similar traits. In the case of gene families regulating innate immunity in bivalves, although some families have ancestrally conserved duplications across bivalve lineages, the expansion of specific families is identified as lineage or species specific.^46^ And regarding the shell and byssus of bivalves, despite the conservation in the morphology, the molecular components of these structures have diverged across bivalve lineages, indicating independent evolution in multiple gene families related to shell formation and byssus fabrication.^51^^,^^59^^,^^60^ Therefore, despite their limited TIMP family, it is speculated that Heteroconchia bivalves may have evolved specific alternative genes to achieve functions related to adaptive traits similar to those carried out by Pteriomorphia TIMPs. For other stationary filter-feeding animals beyond bivalves, the reasons for not experiencing TIMP expansion may follow a similar pattern. One example comes from the barnacles, which are sessile arthropods and have undergone convergent evolution with bivalves. A comparative genomics analysis in barnacles and Pteriomorphia bivalves revealed genome-wide substantial convergent molecular evolution between these two groups. However, there are still multiple divergences in the genetic mechanisms related to convergent adaptive traits, including shell formation and substrate settlement.^57^ The results of this study indicate that barnacles have much less TIMP members than Pteriomorphia bivalves. These suggest that, in the convergent evolution of barnacles and Pteriomorphia bivalves, the TIMP families in these two groups have divergent evolutionary processes. In brief, diverse functions of large TIMP families contribute to some adaptive traits in Pteriomorphia bivalves, while other animals may achieve similar functions through distinct genetic mechanisms. This divergence is hypothesized to be caused by the initial duplication of the TIMP family in Pteriomorphia bivalves, specific to the latest common ancestor of this bivalve clade. The resulting several TIMP gene copies in the ancestral Pteriomorphia bivalves may have undergone neofunctionalization^61^^,^^62^ and further contribute to the multiple adaptive duplication events of TIMP families in their descendants. More studies are required to compare further the evolution dynamics of the TIMP family in Pteriomorphia bivalves and other animals.

Moreover, our findings reveal the widespread presence of pseudo TIMPs in metazoans, which lack conserved NTR domains compared to canonical TIMPs. These pseudo TIMPs exhibit the most extensive repertoire and diversity in bivalves, reaching unprecedented sizes in two Heteroconchia bivalves (18 copies in *D*. *polymorpha*, 11 copies in *M*. *mercenaria*). Among the pseudo TIMPs in molluscs, the majority of pseudo TIMPs in Pteriomorphia bivalves were found grouped within the same orthology groups as canonical TIMPs, suggesting a coeval origin with the multiple independent duplications that contribute to the extensive TIMP repertoire in these bivalves, as discussed earlier. In these cases, the pseudo TIMPs may have emerged through gene duplication events of TIMP, resulting in excess copies, which subsequently underwent N-terminal structural changes to balance the overall functionality of TIMPs within the organism. Conversely, most pseudo TIMPs in Heteroconchia bivalves and the four pseudo TIMPs found in the gastropod *P*. *canaliculata* belong to the orthology groups that do not contain any canonical TIMP, indicating the existence of specific and independent duplications. The expansion of these pseudo TIMPs may have played specific functional roles within the organism, conferring advantages for the survival of certain species and leading to positive natural selection. One hypothesis is that these multiple pseudo TIMPs partially compensate for the functional deficiencies and losses of canonical TIMPs in certain species, such as Heteroconchia bivalves and the gastropod *P*. *canaliculate*. However, the precise roles of these pseudo TIMPs in these molluscs require further investigation in future studies to be fully understood.

In conclusion, our study utilized a high-quality genome of *P*. *penguin* to investigate the evolutionary dynamics of the TIMP gene family within the Metazoa kingdom. Through comparative genomic analyses, we investigated the distribution of the TIMP family across the animal phylogeny and suggested this family originated in the common ancestor of eumetazoans. Our investigation revealed variations in TIMP gene copy numbers across animal lineages, with a massive expansion occurring in the Pteriomorphia infraclass and peaking in the Ostreoida order. These bivalves also exhibit highly diverse TIMP repertoires, which contribute to their multiple adaptive traits, including an effective immune system, and the formation of bivalved shell and byssus. Moreover, our study unveils the presence of abundant pseudo TIMPs, primarily concentrated in Heteroconchia bivalves, highlighting potential future research directions to uncover the exact functions of these genes. Overall, our study provides valuable insights into the evolution of the TIMP gene family in molluscs, bridging the gap in our understanding of the TIMP gene family evolution across the entire Metazoa kingdom. Further investigations into the diverse functions and evolutionary trajectory within the TIMP gene family in Pteriomorphia bivalves are warranted to deepen our understanding.

### Limitations of the study

This study investigates the distribution of TIMP numbers across various metazoan clades using comparative genomic analyses. The reliability of our findings relies on the quality of the investigated genomes, their annotations, and the quantity of genomes studied in each metazoan clade. We gathered metazoan genomes from sources like NCBI (https://www.ncbi.nlm.nih.gov/) and relevant literature and then assessed genome annotation completeness using the BUSCO score as an index. Despite these efforts, a scarcity of publicly available genomes with highly comprehensive annotations, especially in some clades like the Heteroconchia infraclass within bivalves, potentially limited our research scope. A more thorough exploration of TIMP family distribution across animals awaits the release of future high-quality genomes. Additionally, previous research has established the involvement of TIMP family members in various organismal processes within Pteriomorphia bivalves, like immune responses and shell formation. This study refrains from conducting extra systematic experiments to further validate the functions of multiple and diverse TIMP orthologs in *P*. *penguin* or other Pteriomorphia bivalves. Finally, the sex of *P*. *penguin* individuals utilized for genome and transcriptome sequencing was undetermined, as species within the Pteriidae family are usually protandrous hermaphrodites. The gender does not have an impact on the quality of our genome assembly.

## STAR★Methods

### Key resources table

**Table:** 

REAGENT or RESOURCE	SOURCE	IDENTIFIER
**Deposited data**		

*Pteria penguin* genome	This study	PRJNA782667

**Software and algorithms**		

Illumina Novaseq	Illumina	https://www.illumina.com/systems/sequencing-platforms/novaseq.html
PacBio Sequel system	Pacbio	https://www.pacb.com/technology/hifi-sequencing/sequel-system/
Jellyfish version 2.2.10	Marcais and Kingsford^63^	https://github.com/gmarcais/Jellyfish
Genomescope2 version 2.0	Ranallo-Benavidez et al.^64^	http://qb.cshl.edu/genomescope/genomescope2.0/
NextDenovo version 2.4.0	Hu et al.^65^	https://github.com/Nextomics/NextDenovo
purge_dups version 1.2.5	Guan et al.^66^	https://github.com/dfguan/purge_dups
NextPolish version 1.4.0	Hu et al.^67^	https://github.com/Nextomics/NextPolish
fastp version 0.23.2	Chen et al.^68^	https://github.com/OpenGene/fastp
Juicer version 1.6	Durand et al.^69^	https://github.com/aidenlab/juicer
3D *de novo* assembly pipeline version 190716	Dudchenko et al.^70^	https://github.com/aidenlab/3d-dna
Juicebox version 1.11.08	Durand et al.^69^	https://github.com/aidenlab/Juicebox
Burrows-Wheeler Aligner (BWA) version 0.7.17	Li^71^	https://github.com/lh3/bwa
bcftools version 1.15.1	Danecek et al.^72^	https://github.com/samtools/bcftools
BUSCOs version 5.3.2	Manni et al.^73^	https://busco.ezlab.org/
RepeatModeler version 2.0.3	Flynn et al.^74^	https://www.repeatmasker.org/RepeatModeler/
RepeatMasker version 4.1.2	Tarailo-Graovac and Chen^75^	https://www.repeatmasker.org/RepeatMasker/
Infernal version 1.1.4	Nawrocki et al.^76^	http://eddylab.org/infernal/
BRAKER2 version 2.1.6	Bruna et al.^77^	https://github.com/Gaius-Augustus/BRAKER
Trinity version 2.14.0	Haas et al.^78^	https://github.com/trinityrnaseq/trinityrnaseq/wiki
HISAT2 version 2.2.1	Kim et al.^79^	http://daehwankimlab.github.io/hisat2/
Isoseq3 version 3.7.0	PacificBiosciences	https://github.com/PacificBiosciences/pbbioconda
cDNA_Cupcake version 28.0.0	Magdoll	https://github.com/Magdoll/cDNA_Cupcake
PASApipeline version 2.5.2	Haas et al.^80^	https://github.com/PASApipeline/PASApipeline
EVidenceModeler version 1.1.1	Haas et al.^81^	https://github.com/EVidenceModeler/EVidenceModeler
BLAST version 2.13.0	Camacho et al.^82^	https://blast.ncbi.nlm.nih.gov/Blast.cgi
InterProScan version 5.52	Jones et al.^83^	https://github.com/ebi-pf-team/interproscan
eggNOG-mapper website	Cantalapiedra et al.^84^	http://eggnog-mapper.embl.de/
Orthofinder version 2.5.4	Emms and Kelly^85^	https://github.com/davidemms/OrthoFinder
Phylogenetic Analysis by Maximum Likelihood (PAML) version 4.9	Yang^86^	http://abacus.gene.ucl.ac.uk/software/paml.html
Computational Analysis of gene Family Evolution (CAFE) pipeline version 5.1	Mendes et al.^87^	https://github.com/hahnlab/CAFE5
R software version 4.2.1	R Core Team^88^	https://www.r-project.org/
AnnotationForge version 1.42.2	Carlson et al.^89^	https://www.bioconductor.org/packages/AnnotationForge
clusterProfiler 4.0	Wu et al.^90^	https://bioconductor.org/packages/clusterProfiler/
HMMER software version 3.2.1	European Bioinformatics Institute	http://hmmer.org/
IQ-Tree version 2.2.2.6	Minh et al.^91^	http://www.iqtree.org/
SignalP 6.0 website	Teufel et al.^92^	https://services.healthtech.dtu.dk/services/SignalP-6.0/
Adobe Photoshop and Illustrator	Adobe	https://www.adobe.com/
Bioinformatic code for this study	This study	https://github.com/YunweiDongLab/iScience_Chao-yi_Ma

### Resource availability

#### Lead contact

Further information and requests for resources and reagents should be directed to and will be fulfilled by the lead contact, Yun-Wei Dong (dongyw@ouc.edu.cn).

#### Materials availability

This study did not generate new unique reagents.

#### Data and code availability

• The raw genomic sequencing data and *P*. *penguin* genome generated from this study have been deposited in GenBank under the BioProject number PRJNA782667, and are publicly available as of the date of publication. The accession number has been listed in the key resources table.
• All original code has been deposited in the GitHub repository and is publicly available as of the date of publication. The URL link is https://github.com/YunweiDongLab/iScience_Chao-yi_Ma, and has been listed in the key resources table.
• Any additional information required to reanalyze the data reported in this paper is available from the lead contact upon request.

### Experimental model and study participant details

#### The penguin’s wing oyster

A total of three adult penguin’s wing oysters *P*. *penguin* (*Pteria*, Pteriidae) were collected from Wuzhizhou Island, Sanya City, Hainan Province, China, for molecular extraction and sequencing library construction. However, the precise gender of the *P*. *penguin* individuals used in this study was indeterminate, as oysters within the Pteriidae family are usually protandrous hermaphrodites, undergoing successive sex reversals throughout their lifespan in response to various influencing factors.^93^

### Method details

#### Samples preparation and sequencing

Genomic DNA was extracted from the adductor muscle of one adult *P*. *penguin* using the QIAGEN DNeasy Kit (QIAGEN, China), following the manufacturer’s instructions. The genome sequencing was conducted with a combination of long- and short-read technologies. For conducting the genome survey, a paired-end Illumina sequence library with insert size of 350 bp library was prepared and sequenced on an Illumina NovaSeq system (Novogene Co. Ltd., China). The genomic DNA from the same individual was also used for PacBio sequencing library construction, which was then sequenced on a PacBio Sequel II system (Novogene Co. Ltd., China) after the size-selection for enriching DNA fragments longer than 10 kb. The adductor muscle of the same individual was used for Hi-C library construction, in which the tissue was treated with polyformaldehyde (PFA) and the obtained genomic DNA was prepared following a procedure that included cross-linking, digestion by the restriction enzyme DpnII, repairing ends as well as labeling by biotinylated residues. After the genomic DNA was purified and sheared into 350 bp fragments, an Illumina NovaSeq platform (Novogene Co. Ltd., China) was used for subsequent sequencing.

Seven tissues (including adductor muscle, foot, root of foot, gill, labial palps, liver, and mantle) were collected from two adult *P*. *penguin*. Total mRNA was extracted with TRIzol reagent (Invitrogen, USA) based on the manufacturer’s protocol and the assessment of RNA integrity was conducted using the RNA Nano 6000 Assay Kit of the Bioanalyzer 2100 system (Agilent Technologies, USA). To construct sequencing libraries, mRNA was purified from total RNA using poly-T oligo-attached magnetic beads. Then, a total of fourteen Illumina sequencing libraries derived from seven tissues of two adults were sequenced on an Illumina Novaseq system (Novogene Co. Ltd., China) and one Pacbio sequencing library containing all tissues was sequenced on a PacBio Sequel II system (Novogene Co. Ltd., China). Raw reads were filtered by removing low-quality reads and sequencing-adaptor-contaminated reads also were removed; the output data were used for the following genome annotation.

#### Genome survey, assembly, scaffolding and assessment

Genome size, heterozygosity, and repeat content of *P*. *penguin* were estimated by using k-mer analysis. Illumina short reads were first trimmed to remove adaptors and reads with >10% ambiguous or >20% low-quality bases. Then clean reads were used for calculating the distribution of 21-mer frequency with Jellyfish version 2.2.10^63^ and estimating the genome size with Genomescope2 version 2.0.^64^

Contig assembly of *P*. *penguin* using PacBio long reads was conducted via NextDenovo version 2.4.0,^65^ with the parameter “genome_size = 850m”. Both haplotigs and contig overlaps in the primary denovo assembly were identified and removed using purge_dups version 1.2.5^66^ with cutoff values set as “-l 10 -m 110 -u 400”. The non-redundant assembly was subsequently polished by NextPolish version 1.4.0^67^ with Illumina short reads, which had been cleaned via fastp version 0.23.2.^68^

Hi-C sequencing data were used for chromosome-level scaffolding. Polished contigs were first automatically scaffolded using clean Hi-C sequencing reads following the pipeline (https://github.com/esrice/hic-pipeline). The assembly at a scaffold-level was input into Juicer version 1.6^69^ for filtering and deduplicating Hi-C reads. Then, genomic scaffolding was conducted with the 3D *de novo* assembly pipeline version 190716^70^ using the default diploid mode. Several manual corrections were done based on the Hi-C contact maps in Juicebox version 1.11.08^69^ to ensure the scaffolds within the same pseudo chromosomal linkage groups met the Hi-C linkage characteristics. Lastly, 346 contigs were scaffolded into 14 pseudo chromosomal linkage groups, and seven contig debris were produced during the manual correcting process. These debris were not anchored due to insufficient Hi-C linkage found on them. All raw data have been deposited in the database of the National Center for Biotechnology Information (NCBI).

The integrity and accuracy of the genome assembly were evaluated at the single-base level. Illumina short-insert library reads were mapped onto the contigs by using Burrows-Wheeler Aligner (BWA) version 0.7.17^71^ and genetic variants were called out by bcftools version 1.15.1.^72^ Genome completeness was also assessed by using BUSCOs version 5.3.2^73^ and analyzed by searching metazoan gene sets (metazoa_odb10) with genomic mode.

#### Genome annotation

Repetitive sequences in *P*. *penguin* genome assembly were identified and soft-masked prior to gene structure annotation. RepeatModeler version 2.0.3^74^ with option “-LTRStruct” were used to construct a *de novo* canonical database of repetitive elements. Repeat families of Bivalvia were extracted from Repbase (derived RepeatMasker libraries) and Dfam database version 3.2 to build a homology canonical database. These two databases were combined together for identifying and classifying repeats in the *P*. *penguin* genome using RepeatMasker version 4.1.2.^75^ Screens for the genome-wide distribution of noncoding RNA genes were conducted using cmscan program within Infernal software version 1.1.4^76^ with default parameters.

Protein-coding genes were annotated by incorporating ab intio prediction, and transcriptome-assisted methods. Ab intio gene prediction pipeline BRAKER2 version 2.1.6^77^ was used to predict genes with repeat-masked genome sequences by default settings. Transcriptome-assisted annotation was run with Illumina RNA-Seq and Pacbio Iso-Seq data combined. Illumina RNA-Seq data from seven tissues were cleaned via fastp and used for building a comprehensive transcriptome using both *de novo* and genome-based assembly via Trinity version 2.14.0.^78^ For building the genome-based assembly, HISAT2 version 2.2.1^79^ was used to align the clean RNA-Seq data to the *P*. *penguin* genome assembly. Pacbio Iso-seq data were first preprocessed, clustered and filtered to build transcripts structures via Isoseq3 version 3.7.0 (https://github.com/PacificBiosciences/pbbioconda) and cDNA_Cupcake version 28.0.0 (https://github.com/Magdoll/cDNA_Cupcake) following ToFU pipeline.^94^ The *de novo* and genome-guided RNA-Seq assemblies and transcript structures based on Iso-Seq data were inputted to PASA version 2.5.2^80^ following its comprehensive transcriptome database-generating pipeline. Finally, all ab intio gene predictions and transcript alignments were combined into weighted consensus gene structures using EVidenceModeler version 1.1.1.^81^

Functional annotation was performed by homology comparisons of the predicted protein sequences against reviewed SwissProt databases (https://www.uniprot.org/, last modified May 25, 2022) using BLASTP command within BLAST version 2.13.0^82^ with an E-value threshold of 1e-5. Besides, InterProScan version 5.52^83^ and eggNOG-mapper website^84^ was used for gene function and pathway annotation by searching through the Pfam and Gene Ontology (GO) database.

#### Phylogenomic analyses in Mollusca

Full protein sets of eighteen genomes public for use, including seventeen Mollusca species and one Brachiopoda species (*Lingula anatina*), were downloaded and used for phylogenomic analysis as well as orthology inference with *P*. *penguin*. The quality of completeness of full protein sets were evaluated using BUSCOs version 5.3.2 and analyzed by searching metazoan gene sets (metazoa_odb10) with protein mode. And when a gene in each set possessed multiple isoforms, only the longest protein sequence was selected as the representative that was used. Proteomes from all eighteen species were used as the input to Orthofinder version 2.5.4,^85^ which conducted the inference of orthogroups and orthologs, constructed the complete set of gene trees for all orthogroups and the rooted maximum-likelihood species tree. Based on the phylogeny relationship among the species analyzed, the Orthofinder rooted the gene tree for each orthogroup identified, and further conducted a hybrid duplication-loss-coalescence (DLC) algorithm of the rooted gene trees to identify gene duplication events. The hybrid method integrates the advantages of with DLCpar (full) method the highest accuracy,^95^ and the species-overlap method with high efficiency.^96^ MCMCtree,^97^ part of Phylogenetic Analysis by Maximum Likelihood (PAML) version 4.9,^86^ was used to yield the time-calibrated tree, with nodes constrained by fossil records and geographic events. The time frames were used to constrain the nodes in the MCMC tree: minimum = 168.6 Ma and soft maximum = 473.4 Ma for Panpulmonata and Tectipleura;^98^ hard minimum bound = 390 Ma for Caenogastropoda and Heterobranchia;^99^ minimum = 470.2 Ma and soft maximum = 531.5 Ma for *A*. *californica* and *L*. *gigantea*;^98^ hard minimum bound = 419.2 Ma for the first appearance of Mytilidae;^100^^,^^101^ hard minimum = 465.0 Ma for the first appearance of Pteriomorpha;^102^ hard minimum bound = 520 Ma for the first appearance of bivalves;^103^ minimum = 532 Ma and soft maximum = 549 Ma for the first appearance of molluscs;^104^ and minimum = 550.25 Ma and soft maximum = 636.1 Ma for the first appearance of Lophotrochozoan.^104^ The rooted tree from Orthofinder results was used to provide a reference tree topology, and the burn-in, sample frequency, and number of samples were set as 1000000, 10, and 500000, respectively.

#### Orthologous gene family size change analysis in Mollusca

The Orthofinder inferred HOGs, orthogroups at each hierarchical level by analyzing the rooted gene trees for each orthogroups. The dataset containing the species gene number in each HOG was used for the gene family expansion and contraction analysis by Computational Analysis of gene Family Evolution (CAFE) pipeline version 5.1,^87^ and the rooted ultrametric tree from the MCMCtree results were used to provide time-calibrated evolution information. In the calculating process with CAFE 5, the root equilibrium frequency was assumed to be not a uniform distribution and the -p flag is given without a parameter. Gamma model was used to run calculations as if each gene family could belong to a different evolutionary rate category, and *K* = 3–4 gamma rate categories were tested in two independent analyses.^87^ The highest likelihood was found using *K* = 3, with predicted λ = 0.00226 and α = 0.781194, because of its maximum Model Gamma Final Likelihood (-lnL) (254497). The cutoff of the *p*-value was set to 0.05 to determine which families underwent a significant expansion/contraction.

The Gene Ontology (GO) Enrichment Analysis of gene sets in *P*. *penguin* significantly expanded gene families was conducted on R software version 4.2.2.^88^ The R package AnnotationForge^89^ was first used for building a comprehensive annotation data package (org.Ppenguin.eg.db) by incorporating the gene pathway annotation results from InterProScan and eggNOG-mapper website; then the R package clusterProfiler^90^ was used for enrichment analysis.

#### Phylogenetic orthology inference in Metazoa

Full protein sets of forty-one publicly available metazoan genomes (from sixteen Phylum, thirty-nine Class) public for using were downloaded, evaluated using BUSCOs version 5.3.2 analyzed by searching metazoan gene sets (metazoa_odb10) with protein mode, and used for HOGs inference together with *P*. *penguin* by Orthofinder version 2.5.4. The longest protein sequence was selected as the representative that was used when a gene in each set possessed multiple isoforms. To ensure the accuracy of the orthogroups found, the metazoan phylogeny was clarified and a rooted species tree containing the phylogenetic relationship between or within each clade was constructed by referring to multiple studies.^33^^,^^34^^,^^35^^,^^36^^,^^37^^,^^38^^,^^39^^,^^40^

#### TIMP gene family analyses with Mollusca and Metazoa

In two independent analyses, the TIMP gene family was surveyed across the Molluscan and then Metazoan to determine the genomic distribution and calculate the copy numbers. This gene family was first searched from the full protein sets of eighteen molluscs as well as forty-one metazoans mentioned above first using hmmsearch program within HMMER software version 3.2.1 (http://hmmer.org/) with an E-value threshold of 1e^-5^ against a TIMP hmm file (PF00965) from Pfam-A database;^105^ in this procedure, the identical sequences were removed and isoforms coded by one gene (like two sequences in *M*. *gigas*: NP_001292268.1 and XP_011421049.2, coded by the same gene with Gene symbol as LOC105323677) were checked to only keep one. The TIMP sequences were then identified from the sequences obtained using InterProScan;^106^ which searches for protein signature matches, including “METALLOPROTEASE INHIBITOR” in the PANTHER version 17.0, “NTR domain profile” in PROSITE version 2022_05,^107^ and “NTR_2 domain” in SMART version 9.0^108^ databases. Sequences failing to have matches in both the former two protein structure databases were excluded, the remaining were named “putative TIMP” and divided into two groups according to whether matching the conserved NTR domain in the SMART database^109^^,^^110^: one having matches were considered as canonical TIMP, while the other failing to have matches were considered as “pseudo TIMPs”. All of the putative TIMPs were used as inputs for MEME website (https://meme-suite.org/meme/tools/meme) for protein motif discovery;^111^ and in each run, ten motifs were set and expected to be found. According to the accession ID of each TIMP protein, the gene structure information was derived from the genome annotation files in Gff3 format; the whole length and number of exons in each TIMP gene were calculated.

For the phylogenetic analyses of molluscan and metazoan TIMP gene family, the putative TIMP sequences (144 sequences in molluscan TIMP gene family and 114 sequences in the metazoan) and the canonical TIMP sequences from core Mollusca-Brachiopoda TIMP HOG (53 sequences) and core Eumetazoa TIMP HOG (114 sequences) were aligned in Mafft version 7.505^112^ using strategy “L-INS-i”, both the original Mafft alignment of putative sequences and sequences from the two core HOGs were put into IQ-Tree version 2.2.2.6^91^ for Maximum Likelihood (ML) tree construction. In each run, branch supports were evaluated using both 1000 SH-aLRT bootstrap replicates^113^ and 10000 ultrafast bootstrap replicates;^114^ and also optimized using a hill-climbing nearest neighbor interchange (NNI) search by adding “-bnni” flag. For selecting the best model, the ModelFinder contained with IQ-Tree was used,^115^ which identified the best-fitting model as “VT+R6” and “VT+R4” for ML trees constructed from the alignments of putative and core Mollusca-Brachiopoda TIMPs, as “LG+R7” and “LG+R6” for trees constructed from the alignments of putative and core Eumetazoa TIMPs, according to their BIC values. Signal peptides in these two groups of core TIMPs were predicted using SignalP 6.0 program.^92^

### Quantification and statistical analysis

All statistical tests were performed with R (version 4.2.2).

## Supplemental information

Document S1. Figures S1–S8

Table S1. Pteria penguin genome sequencing data information, related to the STAR Methods

Table S2. Pteria penguin 14 pseudomolecules in the genome assembly, related to Figures 1A and S3

Table S3. Summary statistics of the assembled Pteria penguin genome, related to Figure 1A

Table S4. Classification and composition of repeat content in the Pteria penguin genome, related to the STAR Methods

Table S5. Pteria penguin transcriptome sequencing data information, related to the STAR Methods

Table S6. Classification and composition of noncoding RNAs (ncRNAs) in the Pteria penguin genome, related to the STAR Methods

Table S7. The seventeen mollusks and one brachiopod used for phylogenetic orthology inference in this research are related to Figures 1B, 2B, and 4, and the STAR Methods

Table S8. GO categories revealed by enrichment analysis based on the genes from expanded family in the Ostreida clade, related to Figure 2A

Table S9. From the seventeen molluscs and one brachiopod, the sequences identified as putative TIMPs or from the same HOG as putative TIMPs, related to Figures 2B, S2, S4, and S5

Table S10. All the gene duplication events that canonical and pseudo TIMP genes participate in, identified from the seventeen molluscs and one brachiopod by examining each node of each orthogroup gene tree, related to Figures 2B, 4, and S2

Table S11. The forty-one metazoan species used for phylogenetic orthology inference in this research, related to Figures 3 and 4 and the STAR Methods

Table S12. From the forty-one metazoan species, the sequences identified as putative TIMPs or from the same HOG as putative TIMPs, related to Figures 3 and S6–S8

Table S13. All the gene duplication events that canonical and pseudo TIMP genes participate in, identified from the forty-one metazoan species by examining each node of each orthogroup gene tree, related to Figures 3, 4, and S6
