# Supplementary material for: Tracing the evolution of tissue inhibitor of metalloproteinases in Metazoa with the Pteria penguin genome
Source: iScience. 2023 Nov 25;27(1):108579. doi: 10.1016/j.isci.2023.108579 (PMC10755359; doi:10.1016/j.isci.2023.108579)
Supplement: Document S1. Figures S1–S8 [file mmc1.pdf]

iScience, Volume *27*

Supplemental information

Tracing the evolution of tissue inhibitor of metalloproteinases in Metazoa with the *Pteria penguin* genome

Chao-Yi Ma, Yi Chen, Xin Zhan, and Yun-Wei Dong

Supplementary Figure Title and Legends

Figure S1 21 *k*-mer distribution plot for estimating the genome size and heterozygosity of *P. penguin*, related to the STAR Methods.

Figure S2 The phylogenetic trees inferred for each orthogroup with 4 or more sequences, which includes canonical or pseudo TIMP orthologs from seventeen molluscs and one brachiopod. This figure is related to Figure 2. The branches marked with blue denote core Mollusca-Brachiopoda TIMPs, with red denote branch TIMPs, with purple denote pseudo TIMPs, with grey denote sequences coded from the same gene as some of the above TIMPs, and with green denote one sequence isn't identified as canonical or pseudo TIMPs but belonging to the same orthogroup with TIMPs.

Figure S3 Distribution of putative TIMPs in *P. penguin* (upper part) and *M. gigas* (lower part) genome, related to the STAR Methods. The canonical TIMPs, divided into core Mollusca-Brachiopoda TIMPs and branch TIMPs, are respectively marked with blue circles and red triangles; while the pseudo TIMPs are marked with purple boxes.

Figure S4 The phylogenetic relationship of 144 putative TIMPs from seventeen molluscs and one brachiopod including the core Mollusca-Brachiopoda TIMPs (marked in blue), the branch TIMPs (marked in red) pseudo TIMPs (marked in purple). This figure is related to Figure 2. The sizes of orange circles in the tree node denoted the bootstrap support values. From top to bottom, the first ten bands denote the sequence motifs predicted in putative TIMPs via MEME, sorting according to the numbers of each identified motif; the colored boxes represent the presence of motifs while the white boxes represent the absence. The sequence logos of these motifs are demonstrated on the left. The eleventh band marked with one asterisk "*" denotes the whether the NTR domain presents in that sequence (red denotes presence while purple denotes absence). The twelfth and thirteenth band (marked with ** and ***) denote the length of the exons and the exon numbers of TIMP genes, also the deeper color denotes the higher values.

Figure S5 The phylogenetic relationship of 53 core Mollusca-Brachiopoda TIMPs from seventeen molluscs and one brachiopod, related to Figure 2. The sequence motifs predicted by MEME as well as their arrangement order in the sequences are marked with different colors corresponding to those in Figure S4. Signal peptides and NTR domain in these TIMPs are demonstrated.

Figure S6 The phylogenetic trees inferred for each orthogroup with 4 or more sequences, which includes canonical or pseudo TIMP orthologs from forty-one metazoan species. This figure is related to Figure 3. The branches marked with blue denote core Eumetazoa TIMPs, with red denote branch TIMPs, with purple denote pseudo TIMPs, with grey denote sequences coded from the same gene as some of the above TIMPs, and with green denote one sequence isn't identified as canonical or pseudo TIMPs but belonging to the same orthogroup with TIMPs.

Figure S7 The phylogenetic relationship of 165 putative TIMPs from forty-one metazoan species, including the core Eumetazoa TIMPs (marked in blue), the branch TIMPs (marked in red) pseudo TIMPs (marked in purple). This figure is related to Figure 3. The sizes of orange circles in the tree node denoted the bootstrap support values. From top to bottom, the first ten bands denote the motifs predicted in putative TIMPs via MEME, sorting according to the numbers of each identified motif; the colored boxes represent the presence of motifs while the white boxes represent the absence. The sequence logos of these motifs are demonstrated on the left. The eleventh band marked with one asterisk "*" denotes the whether the NTR domain presents in that sequence (red denotes presence while purple denotes absence). The twelfth and thirteenth band (marked with ** and ***) denote the length of the exons and the exon numbers of TIMP genes, also the deeper color denotes the higher values.

Figure S8 The phylogenetic relationship of 114 core Eumetazoa TIMPs from forty-one metazoan species, related to Figure 3. The sequence motifs predicted by MEME as well as their arrangement order in the sequences are marked with corresponding colors in Figure S7. Signal peptides and NTR domain in these TIMPs are demonstrated.

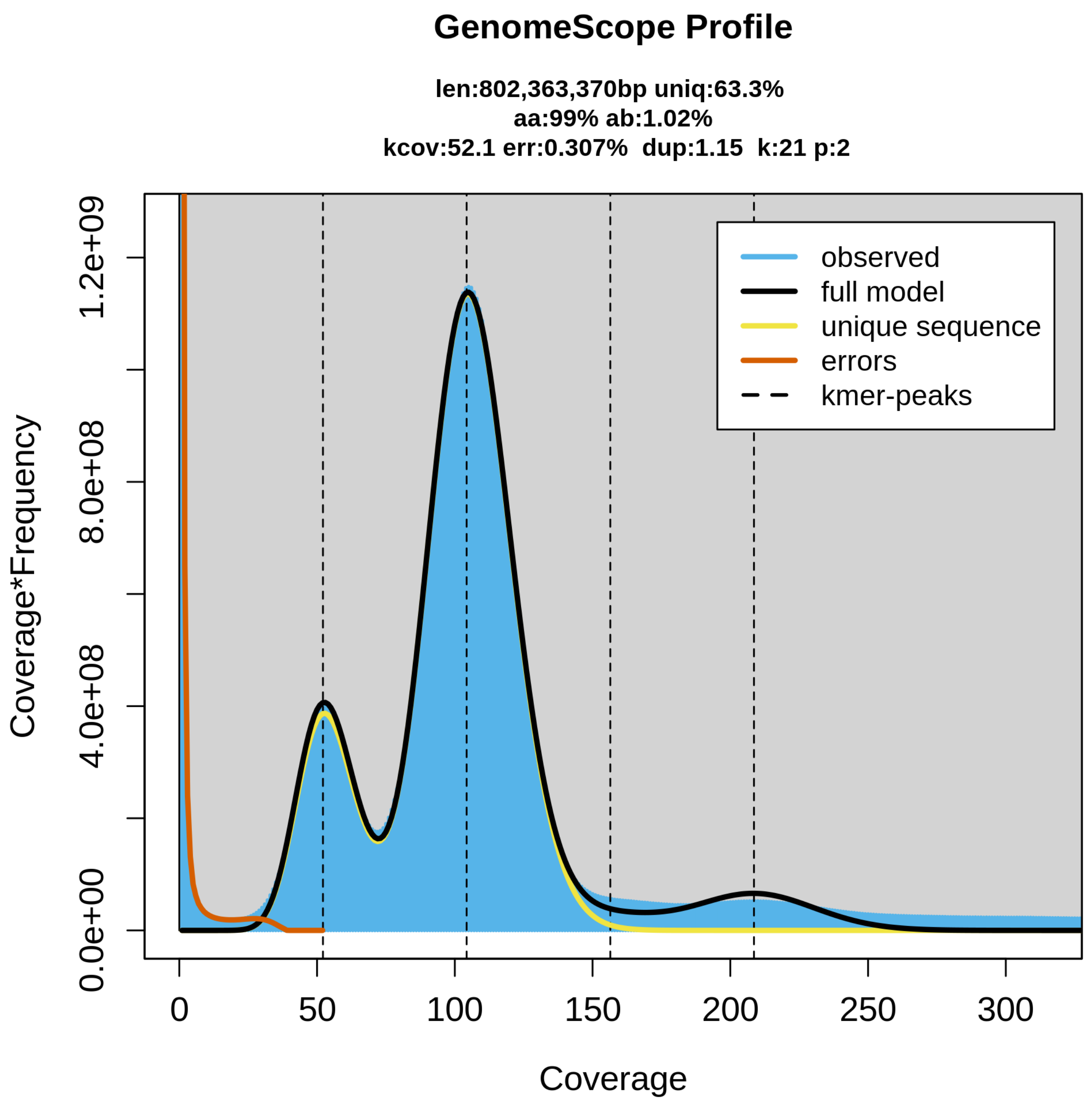
GenomeScope Profile
len:802,363,370bp uniq:63.3%
aa:99% ab:1.02%
kcov:52.1 err:0.307% dup:1.15 k:21 p:2
observed
full model
unique sequence
errors
kmer-peaks
Coverage*Frequency
0.0e+00
4.0e+08
8.0e+08
1.2e+09
0
50
100
150
200
250
300
Coverage

Figure S1

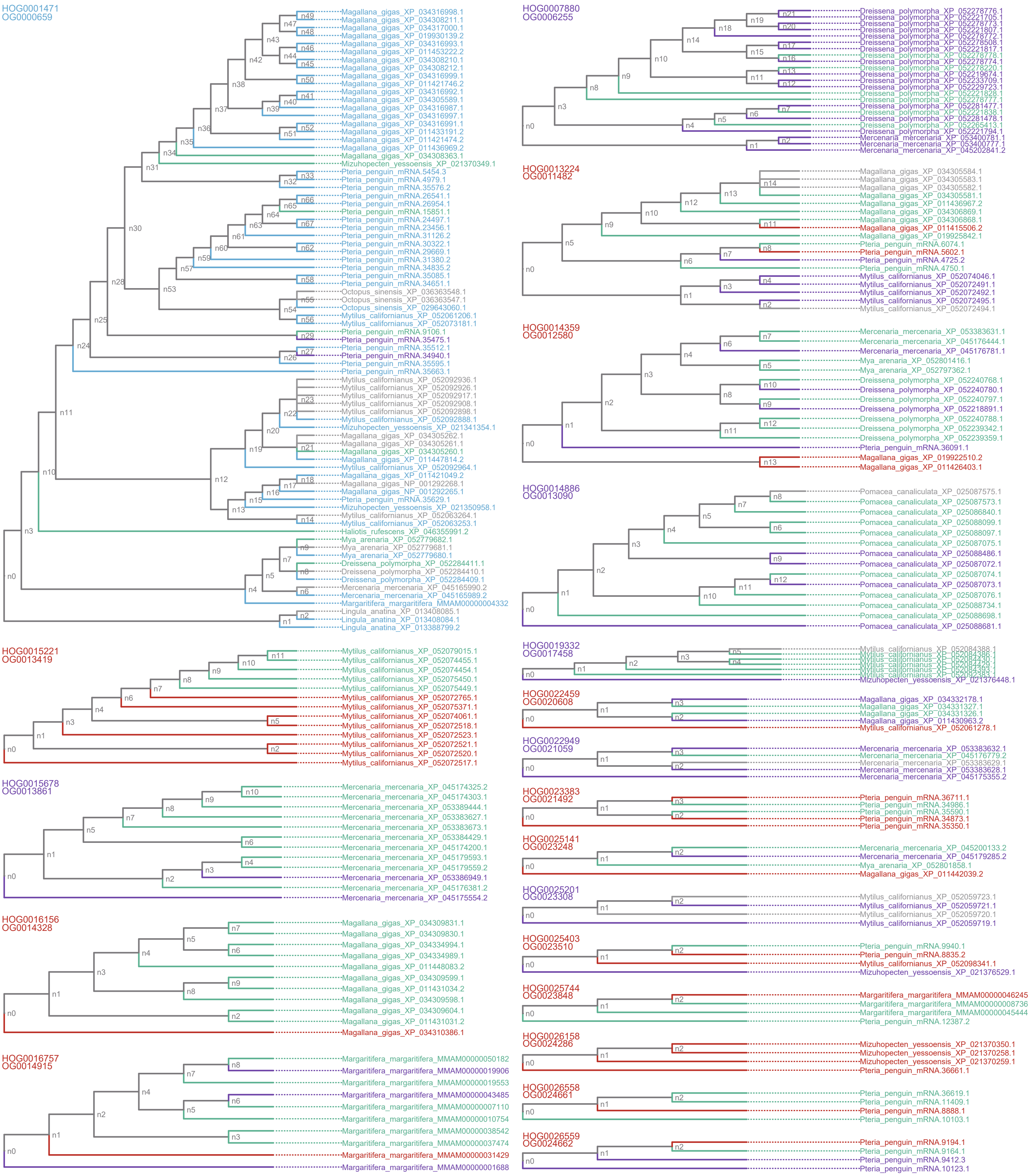
HOG0001471
OG0000659
Magallana_gigas_XP_034316998.1
Magallana_gigas_XP_034308211.1
Magallana_gigas_XP_034317000.1
Magallana_gigas_XP_019930139.2
Magallana_gigas_XP_034316993.1
Magallana_gigas_XP_011453222.2
Magallana_gigas_XP_034308210.1
Magallana_gigas_XP_034308212.1
Magallana_gigas_XP_034316999.1
Magallana_gigas_XP_011421746.2
Magallana_gigas_XP_034316992.1
Magallana_gigas_XP_034305589.1
Magallana_gigas_XP_034316987.1
Magallana_gigas_XP_034316997.1
Magallana_gigas_XP_034316991.1
Magallana_gigas_XP_011433191.2
Magallana_gigas_XP_011421474.2
Magallana_gigas_XP_011436969.2
Magallana_gigas_XP_034308363.1
Mizuhopecten_yessoensis_XP_021370349.1
Pteria_penguin_mRNA.5454.3
Pteria_penguin_mRNA.4979.1
Pteria_penguin_mRNA.35576.2
Pteria_penguin_mRNA.26541.1
Pteria_penguin_mRNA.26954.1
Pteria_penguin_mRNA.15851.1
Pteria_penguin_mRNA.24497.1
Pteria_penguin_mRNA.23456.1
Pteria_penguin_mRNA.31126.2
Pteria_penguin_mRNA.30322.1
Pteria_penguin_mRNA.29669.1
Pteria_penguin_mRNA.31380.2
Pteria_penguin_mRNA.34835.2
Pteria_penguin_mRNA.35085.1
Pteria_penguin_mRNA.34651.1
Octopus_sinensis_XP_036363548.1
Octopus_sinensis_XP_036363547.1
Octopus_sinensis_XP_029643060.1
Mytilus_californianus_XP_052061206.1
Mytilus_californianus_XP_052073181.1
Pteria_penguin_mRNA.9106.1
Pteria_penguin_mRNA.35475.1
Pteria_penguin_mRNA.35512.1
Pteria_penguin_mRNA.34940.1
Pteria_penguin_mRNA.35595.1
Pteria_penguin_mRNA.35663.1
Mytilus_californianus_XP_052092936.1
Mytilus_californianus_XP_052092926.1
Mytilus_californianus_XP_052092917.1
Mytilus_californianus_XP_052092908.1
Mytilus_californianus_XP_052092898.1
Mytilus_californianus_XP_052092888.1
Mizuhopecten_yessoensis_XP_021341354.1
Magallana_gigas_XP_034305262.1
Magallana_gigas_XP_034305261.1
Magallana_gigas_XP_034305260.1
Magallana_gigas_XP_011447814.2
Mytilus_californianus_XP_052092964.1
Magallana_gigas_XP_011421049.2
Magallana_gigas_NP_001292268.1
Magallana_gigas_NP_001292265.1
Pteria_penguin_mRNA.35629.1
Mizuhopecten_yessoensis_XP_021350958.1
Mytilus_californianus_XP_052063264.1
Mytilus_californianus_XP_052063253.1
Haliotis_rufescens_XP_046355991.2
Mya_arenaria_XP_052779682.1
Mya_arenaria_XP_052779681.1
Mya_arenaria_XP_052779680.1
Dreissena_polymorpha_XP_052284411.1
Dreissena_polymorpha_XP_052284410.1
Dreissena_polymorpha_XP_052284409.1
Mercenaria_mercenaria_XP_045165990.2
Mercenaria_mercenaria_XP_045165989.2
Margaritifera_margaritifera_MMAM00000004332
Lingula_anatina_XP_013408085.1
Lingula_anatina_XP_013408084.1
Lingula_anatina_XP_013388799.2
HOG0007880
OG0006255
HOG0013224
OG0011482
Magallana_gigas_XP_034305584.1
Magallana_gigas_XP_034305583.1
Magallana_gigas_XP_034305582.1
Magallana_gigas_XP_034305581.1
Magallana_gigas_XP_011436967.2
Magallana_gigas_XP_034306869.1
Magallana_gigas_XP_034306868.1
Magallana_gigas_XP_011415506.2
Magallana_gigas_XP_019925842.1
Pteria_penguin_mRNA.6074.1
Pteria_penguin_mRNA.5602.1
Pteria_penguin_mRNA.4725.2
Pteria_penguin_mRNA.4750.1
Mytilus_californianus_XP_052074046.1
Mytilus_californianus_XP_052072491.1
Mytilus_californianus_XP_052072492.1
Mytilus_californianus_XP_052072495.1
Mytilus_californianus_XP_052072494.1
HOG0014359
OG0012580
Mercenaria_mercenaria_XP_053383631.1
Mercenaria_mercenaria_XP_045176444.1
Mercenaria_mercenaria_XP_045176781.1
Mya_arenaria_XP_052801416.1
Mya_arenaria_XP_052797362.1
Dreissena_polymorpha_XP_052240768.1
Dreissena_polymorpha_XP_052240780.1
Dreissena_polymorpha_XP_052240797.1
Dreissena_polymorpha_XP_052218891.1
Dreissena_polymorpha_XP_052240788.1
Dreissena_polymorpha_XP_052239342.1
Dreissena_polymorpha_XP_052239359.1
Pteria_penguin_mRNA.36091.1
Magallana_gigas_XP_019922510.2
Magallana_gigas_XP_011426403.1
HOG0014886
OG0013090
Pomacea_canaliculata_XP_025087575.1
Pomacea_canaliculata_XP_025087573.1
Pomacea_canaliculata_XP_025086840.1
Pomacea_canaliculata_XP_025088099.1
Pomacea_canaliculata_XP_025088097.1
Pomacea_canaliculata_XP_025087075.1
Pomacea_canaliculata_XP_025088486.1
Pomacea_canaliculata_XP_025087072.1
Pomacea_canaliculata_XP_025087074.1
Pomacea_canaliculata_XP_025087073.1
Pomacea_canaliculata_XP_025087076.1
Pomacea_canaliculata_XP_025088734.1
Pomacea_canaliculata_XP_025088698.1
Pomacea_canaliculata_XP_025088681.1
HOG0015221
OG0013419
Mytilus_californianus_XP_052079015.1
Mytilus_californianus_XP_052074455.1
Mytilus_californianus_XP_052074454.1
Mytilus_californianus_XP_052075450.1
Mytilus_californianus_XP_052075449.1
Mytilus_californianus_XP_052072765.1
Mytilus_californianus_XP_052075371.1
Mytilus_californianus_XP_052074061.1
Mytilus_californianus_XP_052072518.1
Mytilus_californianus_XP_052072523.1
Mytilus_californianus_XP_052072521.1
Mytilus_californianus_XP_052072520.1
Mytilus_californianus_XP_052072517.1
HOG0019332
OG0017458
Mizuhopecten_yessoensis_XP_021376448.1
HOG0022459
OG0020608
Magallana_gigas_XP_034332178.1
Magallana_gigas_XP_034331327.1
Magallana_gigas_XP_034331326.1
Magallana_gigas_XP_011430963.2
Mytilus_californianus_XP_052061278.1
HOG0022949
OG0021059
Mercenaria_mercenaria_XP_053383632.1
Mercenaria_mercenaria_XP_045176779.2
Mercenaria_mercenaria_XP_053383629.1
Mercenaria_mercenaria_XP_053383628.1
Mercenaria_mercenaria_XP_045175355.2
HOG0015678
OG0013861
Mercenaria_mercenaria_XP_045174325.2
Mercenaria_mercenaria_XP_045174303.1
Mercenaria_mercenaria_XP_053389444.1
Mercenaria_mercenaria_XP_053383627.1
Mercenaria_mercenaria_XP_053383673.1
Mercenaria_mercenaria_XP_053384429.1
Mercenaria_mercenaria_XP_045174200.1
Mercenaria_mercenaria_XP_045179593.1
Mercenaria_mercenaria_XP_045179559.2
Mercenaria_mercenaria_XP_053386949.1
Mercenaria_mercenaria_XP_045176381.2
Mercenaria_mercenaria_XP_045175554.2
HOG0023383
OG0021492
Pteria_penguin_mRNA.36711.1
Pteria_penguin_mRNA.34986.1
Pteria_penguin_mRNA.35590.1
Pteria_penguin_mRNA.34873.1
Pteria_penguin_mRNA.35350.1
HOG0025141
OG0023248
Mercenaria_mercenaria_XP_045200133.2
Mercenaria_mercenaria_XP_045179285.2
Mya_arenaria_XP_052801858.1
Magallana_gigas_XP_011442039.2
HOG0025201
OG0023308
Mytilus_californianus_XP_052059723.1
Mytilus_californianus_XP_052059721.1
Mytilus_californianus_XP_052059720.1
Mytilus_californianus_XP_052059719.1
HOG0016156
OG0014328
Magallana_gigas_XP_034309831.1
Magallana_gigas_XP_034309830.1
Magallana_gigas_XP_034334994.1
Magallana_gigas_XP_034334989.1
Magallana_gigas_XP_011448083.2
Magallana_gigas_XP_034309599.1
Magallana_gigas_XP_011431034.2
Magallana_gigas_XP_034309598.1
Magallana_gigas_XP_034309604.1
Magallana_gigas_XP_011431031.2
Magallana_gigas_XP_034310386.1
HOG0025403
OG0023510
Pteria_penguin_mRNA.9940.1
Pteria_penguin_mRNA.8835.2
Mytilus_californianus_XP_052098341.1
Mizuhopecten_yessoensis_XP_021376529.1
HOG0025744
OG0023848
Margaritifera_margaritifera_MMAM00000046245
Margaritifera_margaritifera_MMAM00000008736
Margaritifera_margaritifera_MMAM00000045444
Pteria_penguin_mRNA.12387.2
HOG0016757
OG0014915
Margaritifera_margaritifera_MMAM00000050182
Margaritifera_margaritifera_MMAM00000019906
Margaritifera_margaritifera_MMAM00000019553
Margaritifera_margaritifera_MMAM00000043485
Margaritifera_margaritifera_MMAM00000007110
Margaritifera_margaritifera_MMAM00000010754
Margaritifera_margaritifera_MMAM00000038542
Margaritifera_margaritifera_MMAM00000037474
Margaritifera_margaritifera_MMAM00000031429
Margaritifera_margaritifera_MMAM00000001688
HOG0026158
OG0024286
Mizuhopecten_yessoensis_XP_021370350.1
Mizuhopecten_yessoensis_XP_021370258.1
Mizuhopecten_yessoensis_XP_021370259.1
Pteria_penguin_mRNA.36661.1
HOG0026558
OG0024661
Pteria_penguin_mRNA.36619.1
Pteria_penguin_mRNA.11409.1
Pteria_penguin_mRNA.8888.1
Pteria_penguin_mRNA.10103.1
HOG0026559
OG0024662
Pteria_penguin_mRNA.9194.1
Pteria_penguin_mRNA.9164.1
Pteria_penguin_mRNA.9412.3
Pteria_penguin_mRNA.10123.1

Figure S2

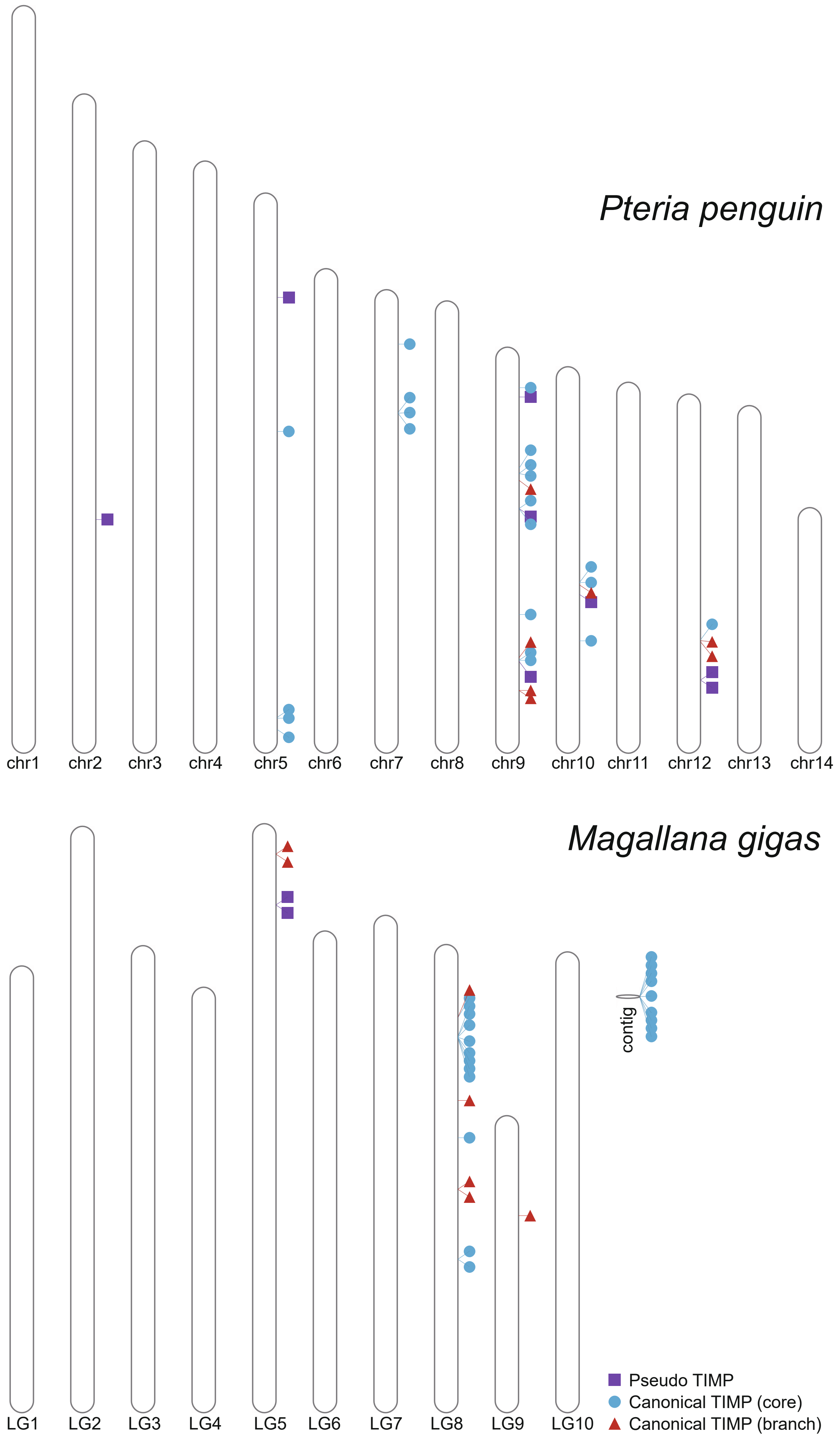

Pteria penguin
chr1
chr2
chr3
chr4
chr5
chr6
chr7
chr8
chr9
chr10
chr11
chr12
chr13
chr14
Magallana gigas
contig
LG1
LG2
LG3
LG4
LG5
LG6
LG7
LG8
LG9
LG10
Pseudo TIMP
Canonical TIMP (core)
Canonical TIMP (branch)

Figure S3

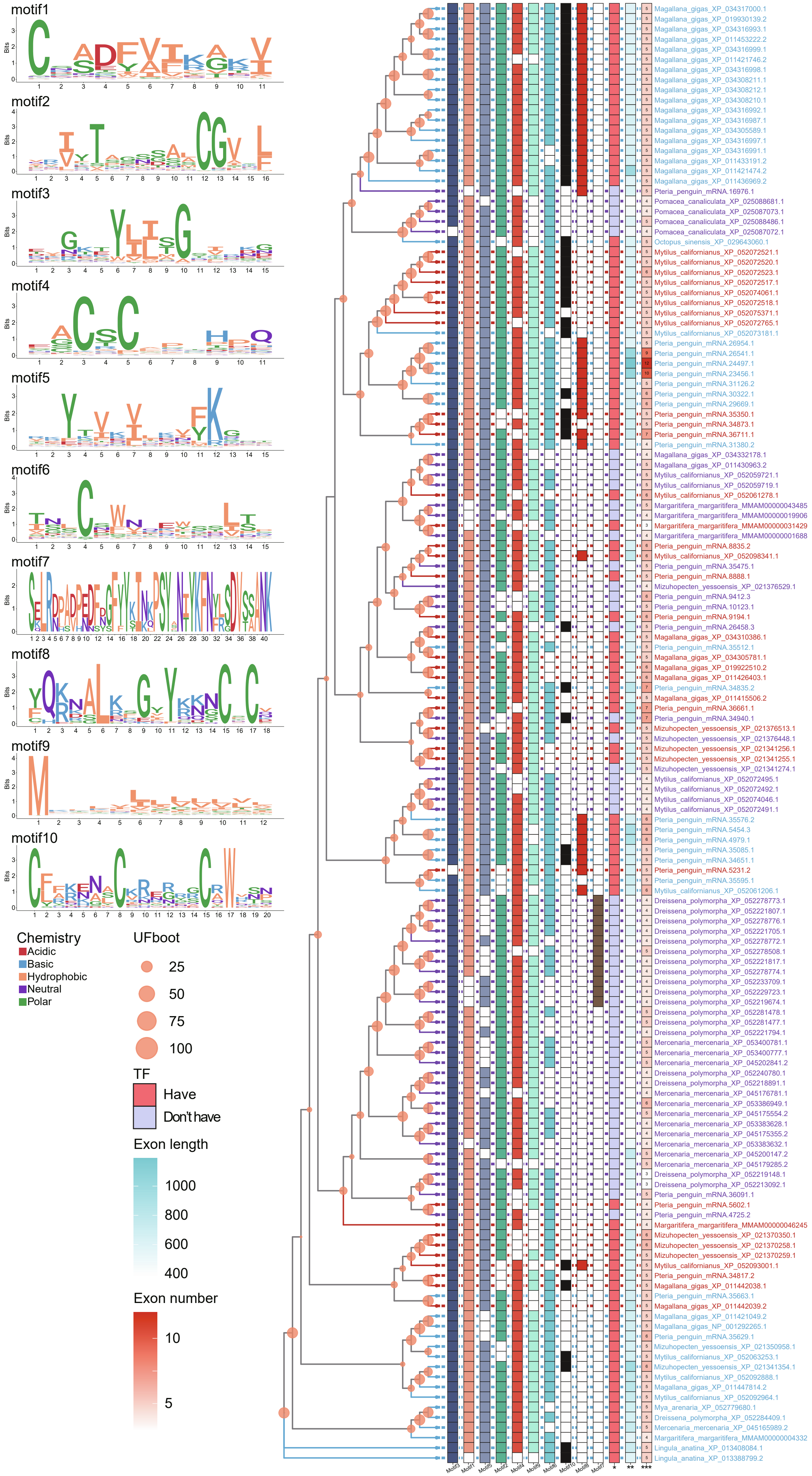
motif1
motif2
motif3
motif4
motif5
motif6
motif7
motif8
motif9
motif10
Bits
Chemistry
Acidic
Basic
Hydrophobic
Neutral
Polar
UFboot
25
50
75
100
TF
Have
Don't have
Exon length
1000
800
600
400
Exon number
10
5
Motif3
Motif1
Motif5
Motif2
Motif4
Motif9
Motif6
Motif10
Motif8
Motif7
*
**
***
Magallana_gigas_XP_034317000.1
Magallana_gigas_XP_019930139.2
Magallana_gigas_XP_034316993.1
Magallana_gigas_XP_011453222.2
Magallana_gigas_XP_034316999.1
Magallana_gigas_XP_011421746.2
Magallana_gigas_XP_034316998.1
Magallana_gigas_XP_034308211.1
Magallana_gigas_XP_034308212.1
Magallana_gigas_XP_034308210.1
Magallana_gigas_XP_034316992.1
Magallana_gigas_XP_034316987.1
Magallana_gigas_XP_034305589.1
Magallana_gigas_XP_034316997.1
Magallana_gigas_XP_034316991.1
Magallana_gigas_XP_011433191.2
Magallana_gigas_XP_011421474.2
Magallana_gigas_XP_011436969.2
Pteria_penguin_mRNA.16976.1
Pomacea_canaliculata_XP_025088681.1
Pomacea_canaliculata_XP_025087073.1
Pomacea_canaliculata_XP_025088486.1
Pomacea_canaliculata_XP_025087072.1
Octopus_sinensis_XP_029643060.1
Mytilus_californianus_XP_052072521.1
Mytilus_californianus_XP_052072520.1
Mytilus_californianus_XP_052072523.1
Mytilus_californianus_XP_052072517.1
Mytilus_californianus_XP_052074061.1
Mytilus_californianus_XP_052072518.1
Mytilus_californianus_XP_052075371.1
Mytilus_californianus_XP_052072765.1
Mytilus_californianus_XP_052073181.1
Pteria_penguin_mRNA.26954.1
Pteria_penguin_mRNA.26541.1
Pteria_penguin_mRNA.24497.1
Pteria_penguin_mRNA.23456.1
Pteria_penguin_mRNA.31126.2
Pteria_penguin_mRNA.30322.1
Pteria_penguin_mRNA.29669.1
Pteria_penguin_mRNA.35350.1
Pteria_penguin_mRNA.34873.1
Pteria_penguin_mRNA.36711.1
Pteria_penguin_mRNA.31380.2
Magallana_gigas_XP_034332178.1
Magallana_gigas_XP_011430963.2
Mytilus_californianus_XP_052059721.1
Mytilus_californianus_XP_052059719.1
Mytilus_californianus_XP_052061278.1
Margaritifera_margaritifera_MMAM00000043485
Margaritifera_margaritifera_MMAM00000019906
Margaritifera_margaritifera_MMAM00000031429
Margaritifera_margaritifera_MMAM00000001688
Pteria_penguin_mRNA.8835.2
Mytilus_californianus_XP_052098341.1
Pteria_penguin_mRNA.35475.1
Pteria_penguin_mRNA.8888.1
Mizuhopecten_yessoensis_XP_021376529.1
Pteria_penguin_mRNA.9412.3
Pteria_penguin_mRNA.10123.1
Pteria_penguin_mRNA.9194.1
Pteria_penguin_mRNA.26458.3
Magallana_gigas_XP_034310386.1
Pteria_penguin_mRNA.35512.1
Magallana_gigas_XP_034305781.1
Magallana_gigas_XP_019922510.2
Magallana_gigas_XP_011426403.1
Pteria_penguin_mRNA.34835.2
Magallana_gigas_XP_011415506.2
Pteria_penguin_mRNA.36661.1
Pteria_penguin_mRNA.34940.1
Mizuhopecten_yessoensis_XP_021376513.1
Mizuhopecten_yessoensis_XP_021376448.1
Mizuhopecten_yessoensis_XP_021341256.1
Mizuhopecten_yessoensis_XP_021341255.1
Mizuhopecten_yessoensis_XP_021341274.1
Mytilus_californianus_XP_052072495.1
Mytilus_californianus_XP_052072492.1
Mytilus_californianus_XP_052074046.1
Mytilus_californianus_XP_052072491.1
Pteria_penguin_mRNA.35576.2
Pteria_penguin_mRNA.5454.3
Pteria_penguin_mRNA.4979.1
Pteria_penguin_mRNA.35085.1
Pteria_penguin_mRNA.34651.1
Pteria_penguin_mRNA.5231.2
Pteria_penguin_mRNA.35595.1
Mytilus_californianus_XP_052061206.1
Dreissena_polymorpha_XP_052278773.1
Dreissena_polymorpha_XP_052221807.1
Dreissena_polymorpha_XP_052278776.1
Dreissena_polymorpha_XP_052221705.1
Dreissena_polymorpha_XP_052278772.1
Dreissena_polymorpha_XP_052278508.1
Dreissena_polymorpha_XP_052221817.1
Dreissena_polymorpha_XP_052278774.1
Dreissena_polymorpha_XP_052233709.1
Dreissena_polymorpha_XP_052229723.1
Dreissena_polymorpha_XP_052219674.1
Dreissena_polymorpha_XP_052281478.1
Dreissena_polymorpha_XP_052281477.1
Dreissena_polymorpha_XP_052221794.1
Mercenaria_mercenaria_XP_053400781.1
Mercenaria_mercenaria_XP_053400777.1
Mercenaria_mercenaria_XP_045202841.2
Dreissena_polymorpha_XP_052240780.1
Dreissena_polymorpha_XP_052218891.1
Mercenaria_mercenaria_XP_045176781.1
Mercenaria_mercenaria_XP_053386949.1
Mercenaria_mercenaria_XP_045175554.2
Mercenaria_mercenaria_XP_053383628.1
Mercenaria_mercenaria_XP_045175355.2
Mercenaria_mercenaria_XP_053383632.1
Mercenaria_mercenaria_XP_045200147.2
Mercenaria_mercenaria_XP_045179285.2
Dreissena_polymorpha_XP_052219148.1
Dreissena_polymorpha_XP_052213092.1
Pteria_penguin_mRNA.36091.1
Pteria_penguin_mRNA.5602.1
Pteria_penguin_mRNA.4725.2
Margaritifera_margaritifera_MMAM00000046245
Mizuhopecten_yessoensis_XP_021370350.1
Mizuhopecten_yessoensis_XP_021370258.1
Mizuhopecten_yessoensis_XP_021370259.1
Mytilus_californianus_XP_052093001.1
Pteria_penguin_mRNA.34817.2
Magallana_gigas_XP_011442038.1
Pteria_penguin_mRNA.35663.1
Magallana_gigas_XP_011442039.2
Magallana_gigas_XP_011421049.2
Magallana_gigas_NP_001292265.1
Pteria_penguin_mRNA.35629.1
Mizuhopecten_yessoensis_XP_021350958.1
Mytilus_californianus_XP_052063253.1
Mizuhopecten_yessoensis_XP_021341354.1
Mytilus_californianus_XP_052092888.1
Magallana_gigas_XP_011447814.2
Mytilus_californianus_XP_052092964.1
Mya_arenaria_XP_052779680.1
Dreissena_polymorpha_XP_052284409.1
Mercenaria_mercenaria_XP_045165989.2
Margaritifera_margaritifera_MMAM00000004332
Lingula_anatina_XP_013408084.1
Lingula_anatina_XP_013388799.2

Figure S4

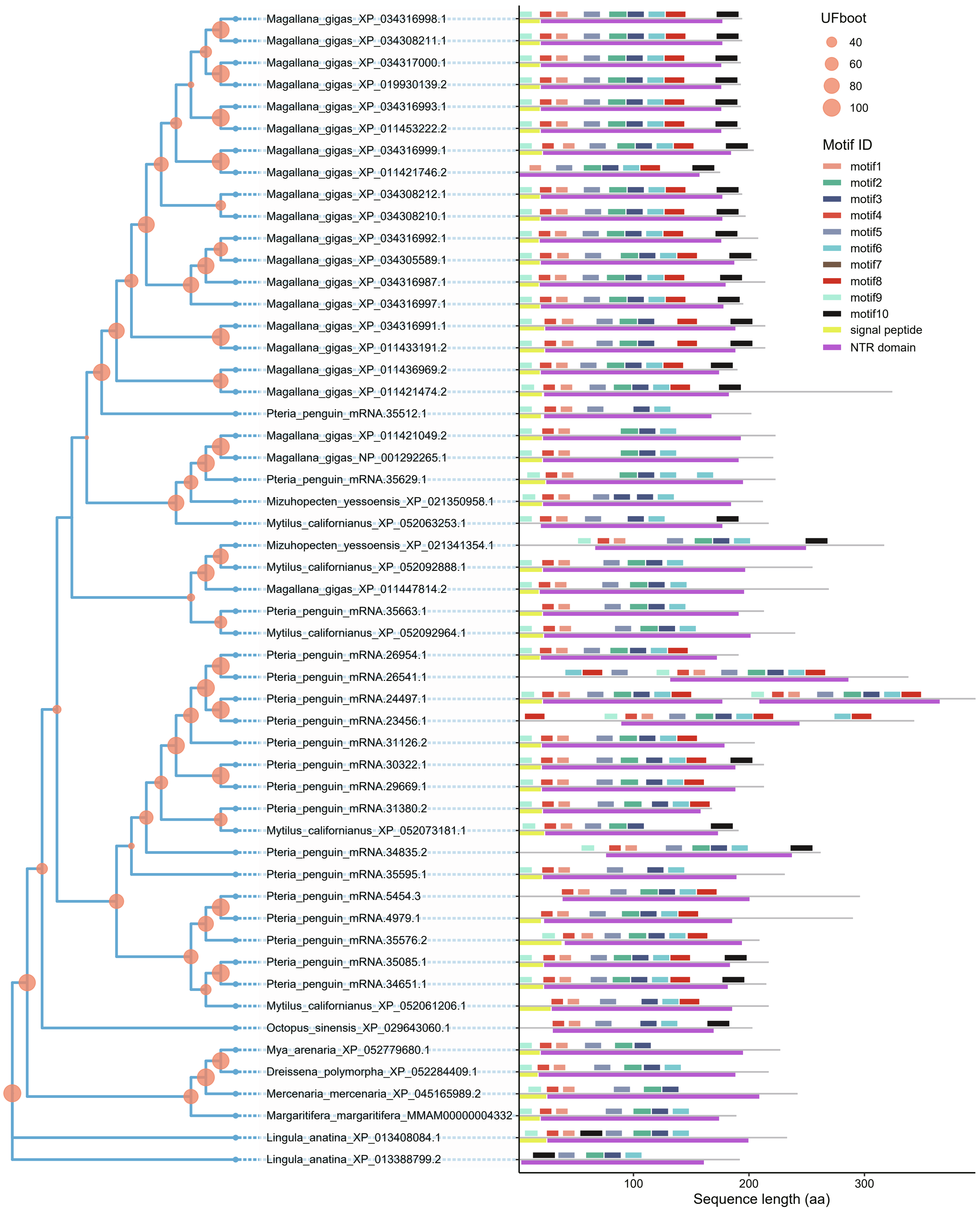
Magallana_gigas_XP_034316998.1
Magallana_gigas_XP_034308211.1
Magallana_gigas_XP_034317000.1
Magallana_gigas_XP_019930139.2
Magallana_gigas_XP_034316993.1
Magallana_gigas_XP_011453222.2
Magallana_gigas_XP_034316999.1
Magallana_gigas_XP_011421746.2
Magallana_gigas_XP_034308212.1
Magallana_gigas_XP_034308210.1
Magallana_gigas_XP_034316992.1
Magallana_gigas_XP_034305589.1
Magallana_gigas_XP_034316987.1
Magallana_gigas_XP_034316997.1
Magallana_gigas_XP_034316991.1
Magallana_gigas_XP_011433191.2
Magallana_gigas_XP_011436969.2
Magallana_gigas_XP_011421474.2
Pteria_penguin_mRNA.35512.1
Magallana_gigas_XP_011421049.2
Magallana_gigas_NP_001292265.1
Pteria_penguin_mRNA.35629.1
Mizuhopecten_yessoensis_XP_021350958.1
Mytilus_californianus_XP_052063253.1
Mizuhopecten_yessoensis_XP_021341354.1
Mytilus_californianus_XP_052092888.1
Magallana_gigas_XP_011447814.2
Pteria_penguin_mRNA.35663.1
Mytilus_californianus_XP_052092964.1
Pteria_penguin_mRNA.26954.1
Pteria_penguin_mRNA.26541.1
Pteria_penguin_mRNA.24497.1
Pteria_penguin_mRNA.23456.1
Pteria_penguin_mRNA.31126.2
Pteria_penguin_mRNA.30322.1
Pteria_penguin_mRNA.29669.1
Pteria_penguin_mRNA.31380.2
Mytilus_californianus_XP_052073181.1
Pteria_penguin_mRNA.34835.2
Pteria_penguin_mRNA.35595.1
Pteria_penguin_mRNA.5454.3
Pteria_penguin_mRNA.4979.1
Pteria_penguin_mRNA.35576.2
Pteria_penguin_mRNA.35085.1
Pteria_penguin_mRNA.34651.1
Mytilus_californianus_XP_052061206.1
Octopus_sinensis_XP_029643060.1
Mya_arenaria_XP_052779680.1
Dreissena_polymorpha_XP_052284409.1
Mercenaria_mercenaria_XP_045165989.2
Margaritifera_margaritifera_MMAM00000004332
Lingula_anatina_XP_013408084.1
Lingula_anatina_XP_013388799.2
UFboot
40
60
80
100
Motif ID
motif1
motif2
motif3
motif4
motif5
motif6
motif7
motif8
motif9
motif10
signal peptide
NTR domain
100
200
300
Sequence length (aa)

Figure S5

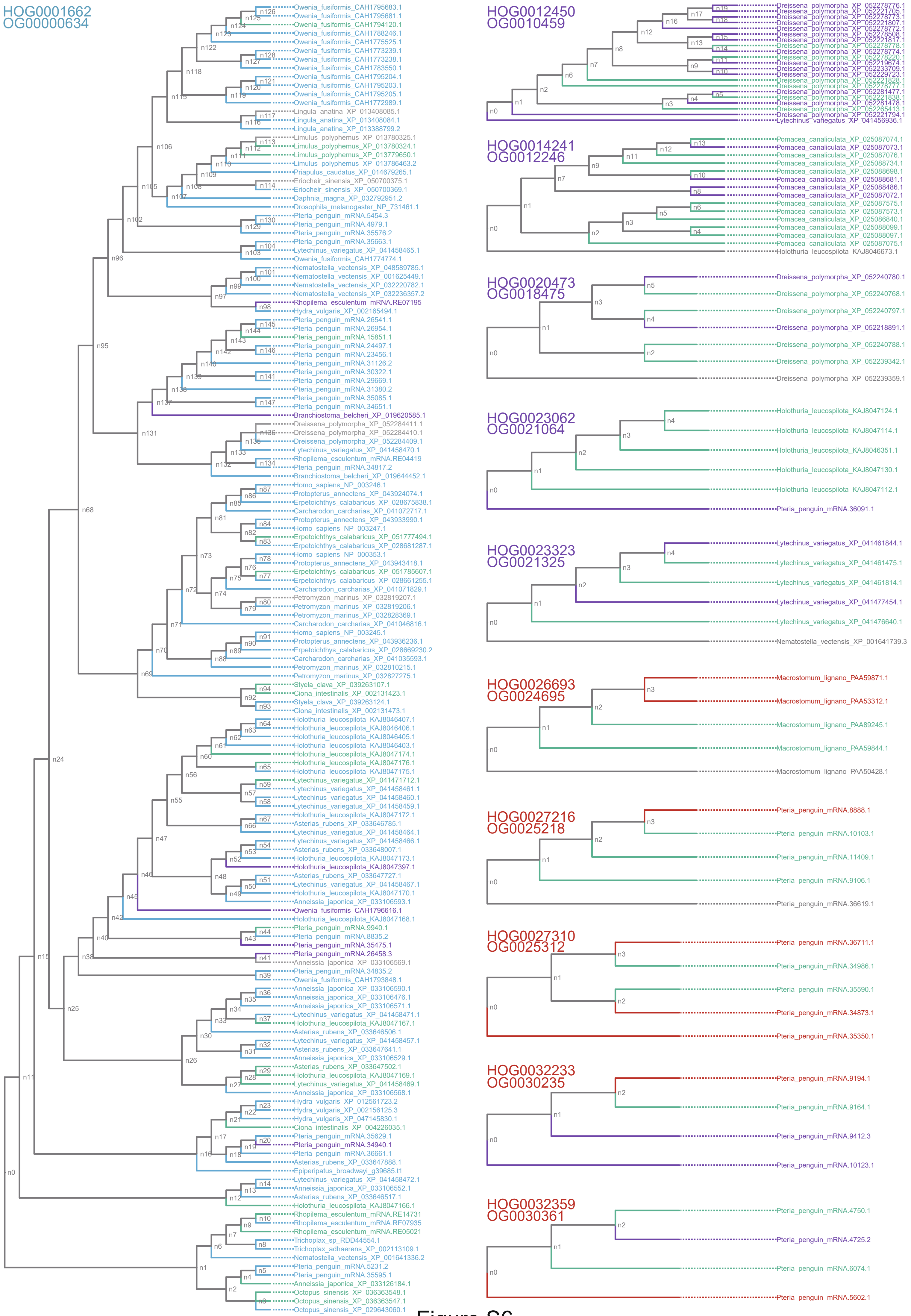
HOG0001662
OG0000634
Owenia_fusiformis_CAH1795683.1
Owenia_fusiformis_CAH1795681.1
Owenia_fusiformis_CAH1794120.1
Owenia_fusiformis_CAH1788246.1
Owenia_fusiformis_CAH1775525.1
Owenia_fusiformis_CAH1773239.1
Owenia_fusiformis_CAH1773238.1
Owenia_fusiformis_CAH1783550.1
Owenia_fusiformis_CAH1795204.1
Owenia_fusiformis_CAH1795203.1
Owenia_fusiformis_CAH1795205.1
Owenia_fusiformis_CAH1772989.1
Lingula_anatina_XP_013408085.1
Lingula_anatina_XP_013408084.1
Lingula_anatina_XP_013388799.2
Limulus_polyphemus_XP_013780325.1
Limulus_polyphemus_XP_013780324.1
Limulus_polyphemus_XP_013779650.1
Limulus_polyphemus_XP_013786463.2
Priapulus_caudatus_XP_014679265.1
Eriocheir_sinensis_XP_050700375.1
Eriocheir_sinensis_XP_050700369.1
Daphnia_magna_XP_032792951.2
Drosophila_melanogaster_NP_731461.1
Pteria_penguin_mRNA.5454.3
Pteria_penguin_mRNA.4979.1
Pteria_penguin_mRNA.35576.2
Pteria_penguin_mRNA.35663.1
Lytechinus_variegatus_XP_041458465.1
Owenia_fusiformis_CAH1774774.1
Nematostella_vectensis_XP_048589785.1
Nematostella_vectensis_XP_001625449.1
Nematostella_vectensis_XP_032220782.1
Nematostella_vectensis_XP_032236357.2
Rhopilema_esculentum_mRNA.RE07195
Hydra_vulgaris_XP_002165494.1
Pteria_penguin_mRNA.26541.1
Pteria_penguin_mRNA.26954.1
Pteria_penguin_mRNA.15851.1
Pteria_penguin_mRNA.24497.1
Pteria_penguin_mRNA.23456.1
Pteria_penguin_mRNA.31126.2
Pteria_penguin_mRNA.30322.1
Pteria_penguin_mRNA.29669.1
Pteria_penguin_mRNA.31380.2
Pteria_penguin_mRNA.35085.1
Pteria_penguin_mRNA.34651.1
Branchiostoma_belcheri_XP_019620585.1
Dreissena_polymorpha_XP_052284411.1
Dreissena_polymorpha_XP_052284410.1
Dreissena_polymorpha_XP_052284409.1
Lytechinus_variegatus_XP_041458470.1
Rhopilema_esculentum_mRNA.RE04419
Pteria_penguin_mRNA.34817.2
Branchiostoma_belcheri_XP_019644452.1
Homo_sapiens_NP_003246.1
Protopterus_annectens_XP_043924074.1
Erpetoichthys_calabaricus_XP_028675838.1
Carcharodon_carcharias_XP_041072717.1
Protopterus_annectens_XP_043933990.1
Homo_sapiens_NP_003247.1
Erpetoichthys_calabaricus_XP_051777494.1
Erpetoichthys_calabaricus_XP_028681287.1
Homo_sapiens_NP_000353.1
Protopterus_annectens_XP_043943418.1
Erpetoichthys_calabaricus_XP_051785607.1
Erpetoichthys_calabaricus_XP_028661255.1
Carcharodon_carcharias_XP_041071829.1
Petromyzon_marinus_XP_032819207.1
Petromyzon_marinus_XP_032819206.1
Petromyzon_marinus_XP_032828369.1
Carcharodon_carcharias_XP_041046816.1
Homo_sapiens_NP_003245.1
Protopterus_annectens_XP_043936236.1
Erpetoichthys_calabaricus_XP_028669230.2
Carcharodon_carcharias_XP_041035593.1
Petromyzon_marinus_XP_032810215.1
Petromyzon_marinus_XP_032827275.1
Styela_clava_XP_039263107.1
Ciona_intestinalis_XP_002131423.1
Styela_clava_XP_039263124.1
Ciona_intestinalis_XP_002131473.1
Holothuria_leucospilota_KAJ8046407.1
Holothuria_leucospilota_KAJ8046406.1
Holothuria_leucospilota_KAJ8046405.1
Holothuria_leucospilota_KAJ8046403.1
Holothuria_leucospilota_KAJ8047174.1
Holothuria_leucospilota_KAJ8047176.1
Holothuria_leucospilota_KAJ8047175.1
Lytechinus_variegatus_XP_041471712.1
Lytechinus_variegatus_XP_041458461.1
Lytechinus_variegatus_XP_041458460.1
Lytechinus_variegatus_XP_041458459.1
Holothuria_leucospilota_KAJ8047172.1
Asterias_rubens_XP_033646785.1
Lytechinus_variegatus_XP_041458464.1
Lytechinus_variegatus_XP_041458466.1
Asterias_rubens_XP_033648007.1
Holothuria_leucospilota_KAJ8047173.1
Holothuria_leucospilota_KAJ8047397.1
Asterias_rubens_XP_033647727.1
Lytechinus_variegatus_XP_041458467.1
Holothuria_leucospilota_KAJ8047170.1
Anneissia_japonica_XP_033106593.1
Owenia_fusiformis_CAH1796616.1
Holothuria_leucospilota_KAJ8047168.1
Pteria_penguin_mRNA.9940.1
Pteria_penguin_mRNA.8835.2
Pteria_penguin_mRNA.35475.1
Pteria_penguin_mRNA.26458.3
Anneissia_japonica_XP_033106569.1
Pteria_penguin_mRNA.34835.2
Owenia_fusiformis_CAH1793848.1
Anneissia_japonica_XP_033106590.1
Anneissia_japonica_XP_033106476.1
Anneissia_japonica_XP_033106571.1
Lytechinus_variegatus_XP_041458471.1
Holothuria_leucospilota_KAJ8047167.1
Asterias_rubens_XP_033646506.1
Lytechinus_variegatus_XP_041458457.1
Asterias_rubens_XP_033647641.1
Anneissia_japonica_XP_033106529.1
Asterias_rubens_XP_033647502.1
Holothuria_leucospilota_KAJ8047169.1
Lytechinus_variegatus_XP_041458469.1
Anneissia_japonica_XP_033106568.1
Hydra_vulgaris_XP_012561723.2
Hydra_vulgaris_XP_002156125.3
Hydra_vulgaris_XP_047145830.1
Ciona_intestinalis_XP_004226035.1
Pteria_penguin_mRNA.35629.1
Pteria_penguin_mRNA.34940.1
Pteria_penguin_mRNA.36661.1
Asterias_rubens_XP_033647888.1
Epiperipatus_broadwayi_g39685.t1
Lytechinus_variegatus_XP_041458472.1
Anneissia_japonica_XP_033106552.1
Asterias_rubens_XP_033646517.1
Holothuria_leucospilota_KAJ8047166.1
Rhopilema_esculentum_mRNA.RE14731
Rhopilema_esculentum_mRNA.RE07935
Rhopilema_esculentum_mRNA.RE05021
Trichoplax_sp_RDD44554.1
Trichoplax_adhaerens_XP_002113109.1
Nematostella_vectensis_XP_001641336.2
Pteria_penguin_mRNA.5231.2
Pteria_penguin_mRNA.35595.1
Anneissia_japonica_XP_033126184.1
Octopus_sinensis_XP_036363548.1
Octopus_sinensis_XP_036363547.1
Octopus_sinensis_XP_029643060.1
HOG0012450
OG0010459
Dreissena_polymorpha_XP_052278776.1
Dreissena_polymorpha_XP_052221705.1
Dreissena_polymorpha_XP_052278773.1
Dreissena_polymorpha_XP_052221807.1
Dreissena_polymorpha_XP_052278772.1
Dreissena_polymorpha_XP_052278508.1
Dreissena_polymorpha_XP_052221817.1
Dreissena_polymorpha_XP_052278778.1
Dreissena_polymorpha_XP_052278774.1
Dreissena_polymorpha_XP_052278220.1
Dreissena_polymorpha_XP_052219674.1
Dreissena_polymorpha_XP_052233709.1
Dreissena_polymorpha_XP_052229723.1
Dreissena_polymorpha_XP_052221828.1
Dreissena_polymorpha_XP_052278777.1
Dreissena_polymorpha_XP_052281477.1
Dreissena_polymorpha_XP_052221838.1
Dreissena_polymorpha_XP_052281478.1
Dreissena_polymorpha_XP_052265413.1
Dreissena_polymorpha_XP_052221794.1
Lytechinus_variegatus_XP_041456936.1
HOG0014241
OG0012246
Pomacea_canaliculata_XP_025087074.1
Pomacea_canaliculata_XP_025087073.1
Pomacea_canaliculata_XP_025087076.1
Pomacea_canaliculata_XP_025088734.1
Pomacea_canaliculata_XP_025088698.1
Pomacea_canaliculata_XP_025088681.1
Pomacea_canaliculata_XP_025088486.1
Pomacea_canaliculata_XP_025087072.1
Pomacea_canaliculata_XP_025087575.1
Pomacea_canaliculata_XP_025087573.1
Pomacea_canaliculata_XP_025086840.1
Pomacea_canaliculata_XP_025088099.1
Pomacea_canaliculata_XP_025088097.1
Pomacea_canaliculata_XP_025087075.1
Holothuria_leucospilota_KAJ8046673.1
HOG0020473
OG0018475
Dreissena_polymorpha_XP_052240780.1
Dreissena_polymorpha_XP_052240768.1
Dreissena_polymorpha_XP_052240797.1
Dreissena_polymorpha_XP_052218891.1
Dreissena_polymorpha_XP_052240788.1
Dreissena_polymorpha_XP_052239342.1
Dreissena_polymorpha_XP_052239359.1
HOG0023062
OG0021064
Holothuria_leucospilota_KAJ8047124.1
Holothuria_leucospilota_KAJ8047114.1
Holothuria_leucospilota_KAJ8046351.1
Holothuria_leucospilota_KAJ8047130.1
Holothuria_leucospilota_KAJ8047112.1
Pteria_penguin_mRNA.36091.1
HOG0023323
OG0021325
Lytechinus_variegatus_XP_041461844.1
Lytechinus_variegatus_XP_041461475.1
Lytechinus_variegatus_XP_041461814.1
Lytechinus_variegatus_XP_041477454.1
Lytechinus_variegatus_XP_041476640.1
Nematostella_vectensis_XP_001641739.3
HOG0026693
OG0024695
Macrostomum_lignano_PAA59871.1
Macrostomum_lignano_PAA53312.1
Macrostomum_lignano_PAA89245.1
Macrostomum_lignano_PAA59844.1
Macrostomum_lignano_PAA50428.1
HOG0027216
OG0025218
Pteria_penguin_mRNA.8888.1
Pteria_penguin_mRNA.10103.1
Pteria_penguin_mRNA.11409.1
Pteria_penguin_mRNA.9106.1
Pteria_penguin_mRNA.36619.1
HOG0027310
OG0025312
Pteria_penguin_mRNA.36711.1
Pteria_penguin_mRNA.34986.1
Pteria_penguin_mRNA.35590.1
Pteria_penguin_mRNA.34873.1
Pteria_penguin_mRNA.35350.1
HOG0032233
OG0030235
Pteria_penguin_mRNA.9194.1
Pteria_penguin_mRNA.9164.1
Pteria_penguin_mRNA.9412.3
Pteria_penguin_mRNA.10123.1
HOG0032359
OG0030361
Pteria_penguin_mRNA.4750.1
Pteria_penguin_mRNA.4725.2
Pteria_penguin_mRNA.6074.1
Pteria_penguin_mRNA.5602.1

Figure S6

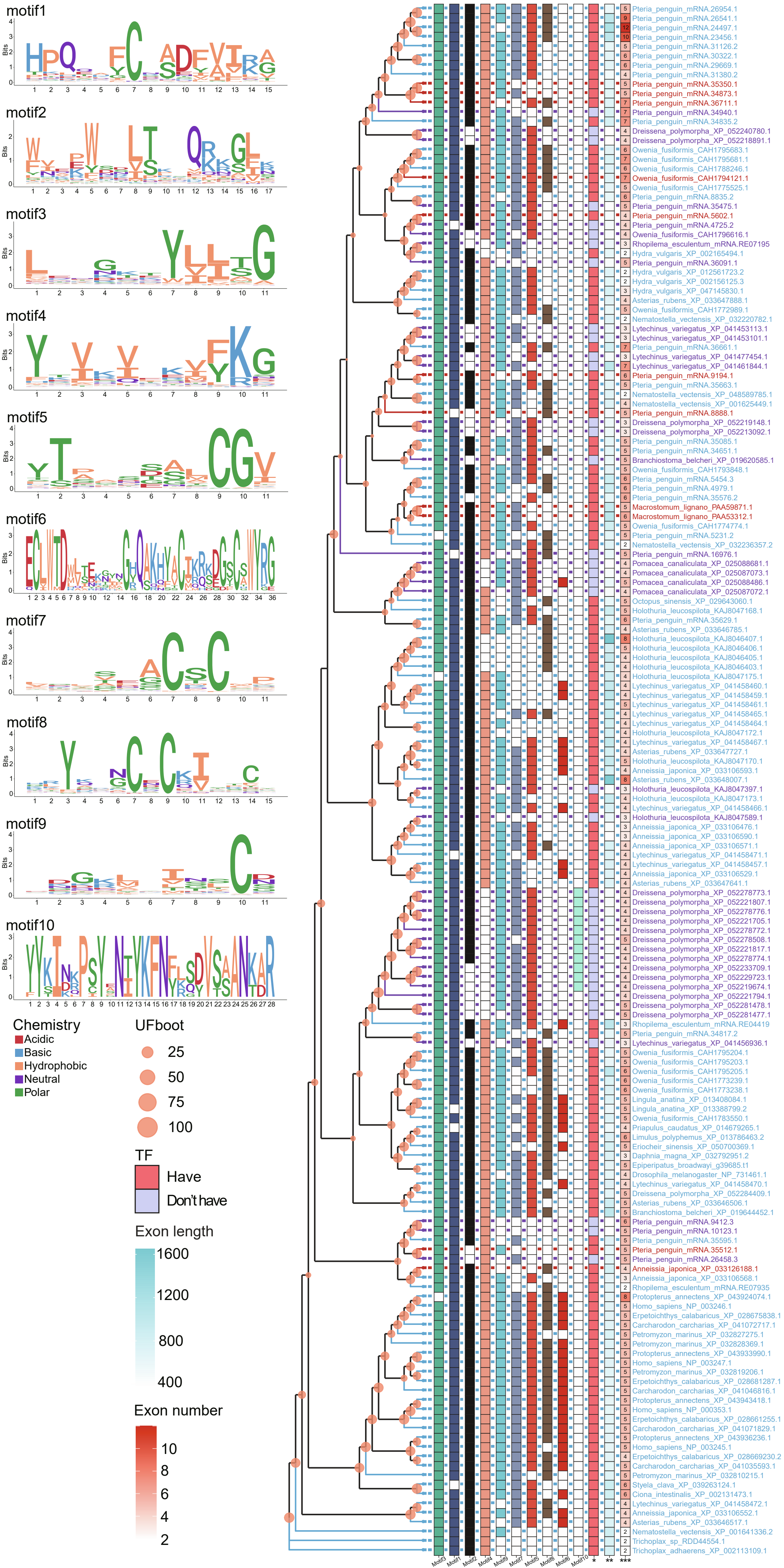
motif1
motif2
motif3
motif4
motif5
motif6
motif7
motif8
motif9
motif10
Bits
Chemistry
Acidic
Basic
Hydrophobic
Neutral
Polar
UFboot
25
50
75
100
TF
Have
Don't have
Exon length
1600
1200
800
400
Exon number
10
8
6
4
2
Motif3
Motif1
Motif2
Motif4
Motif9
Motif7
Motif5
Motif8
Motif6
Motif10
*
**
***

Figure S7

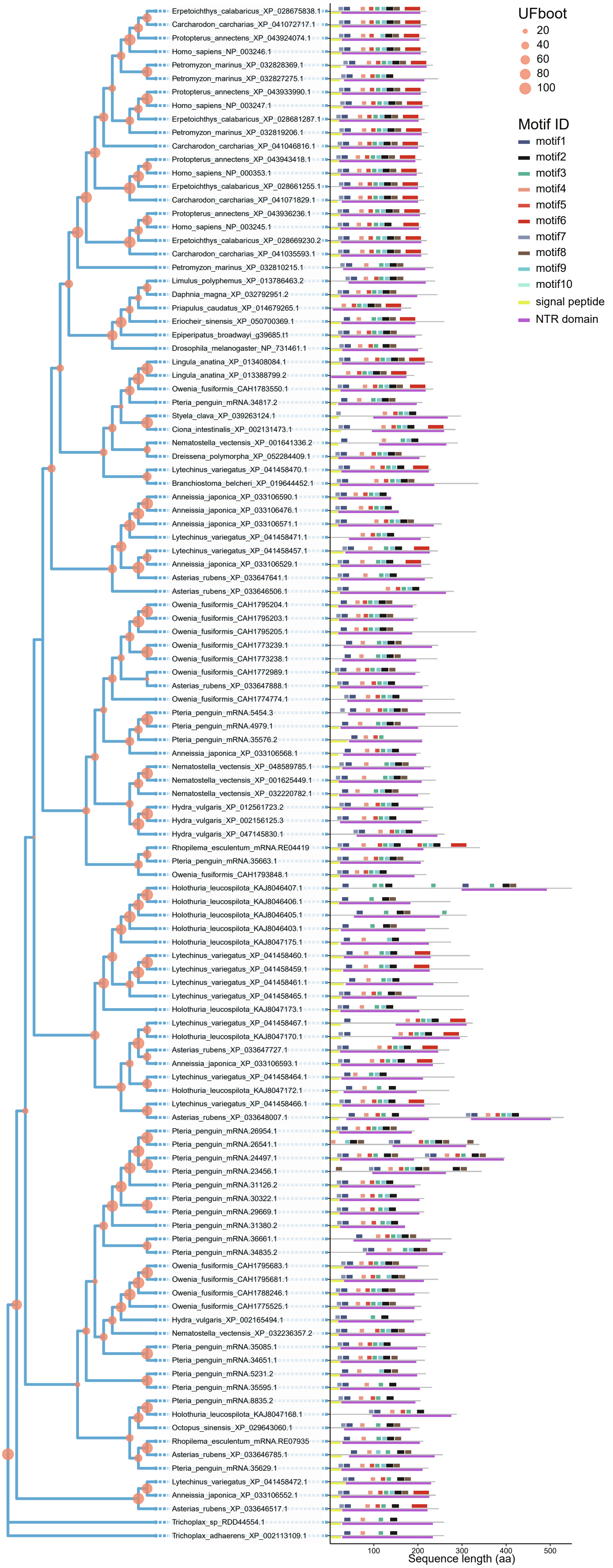

Erpetoichthys_calabaricus_XP_028675838.1
Carcharodon_carcharias_XP_041072717.1
Protopterus_annectens_XP_043924074.1
Homo_sapiens_NP_003246.1
Petromyzon_marinus_XP_032828369.1
Petromyzon_marinus_XP_032827275.1
Protopterus_annectens_XP_043933990.1
Homo_sapiens_NP_003247.1
Erpetoichthys_calabaricus_XP_028681287.1
Petromyzon_marinus_XP_032819206.1
Carcharodon_carcharias_XP_041046816.1
Protopterus_annectens_XP_043943418.1
Homo_sapiens_NP_000353.1
Erpetoichthys_calabaricus_XP_028661255.1
Carcharodon_carcharias_XP_041071829.1
Protopterus_annectens_XP_043936236.1
Homo_sapiens_NP_003245.1
Erpetoichthys_calabaricus_XP_028669230.2
Carcharodon_carcharias_XP_041035593.1
Petromyzon_marinus_XP_032810215.1
Limulus_polyphemus_XP_013786463.2
Daphnia_magna_XP_032792951.2
Priapulus_caudatus_XP_014679265.1
Eriocheir_sinensis_XP_050700369.1
Epiperipatus_broadwayi_g39685.t1
Drosophila_melanogaster_NP_731461.1
Lingula_anatina_XP_013408084.1
Lingula_anatina_XP_013388799.2
Owenia_fusiformis_CAH1783550.1
Pteria_penguin_mRNA.34817.2
Styela_clava_XP_039263124.1
Ciona_intestinalis_XP_002131473.1
Nematostella_vectensis_XP_001641336.2
Dreissena_polymorpha_XP_052284409.1
Lytechinus_variegatus_XP_041458470.1
Branchiostoma_belcheri_XP_019644452.1
Anneissia_japonica_XP_033106590.1
Anneissia_japonica_XP_033106476.1
Anneissia_japonica_XP_033106571.1
Lytechinus_variegatus_XP_041458471.1
Lytechinus_variegatus_XP_041458457.1
Anneissia_japonica_XP_033106529.1
Asterias_rubens_XP_033647641.1
Asterias_rubens_XP_033646506.1
Owenia_fusiformis_CAH1795204.1
Owenia_fusiformis_CAH1795203.1
Owenia_fusiformis_CAH1795205.1
Owenia_fusiformis_CAH1773239.1
Owenia_fusiformis_CAH1773238.1
Owenia_fusiformis_CAH1772989.1
Asterias_rubens_XP_033647888.1
Owenia_fusiformis_CAH1774774.1
Pteria_penguin_mRNA.5454.3
Pteria_penguin_mRNA.4979.1
Pteria_penguin_mRNA.35576.2
Anneissia_japonica_XP_033106568.1
Nematostella_vectensis_XP_048589785.1
Nematostella_vectensis_XP_001625449.1
Nematostella_vectensis_XP_032220782.1
Hydra_vulgaris_XP_012561723.2
Hydra_vulgaris_XP_002156125.3
Hydra_vulgaris_XP_047145830.1
Rhopilema_esculentum_mRNA.RE04419
Pteria_penguin_mRNA.35663.1
Owenia_fusiformis_CAH1793848.1
Holothuria_leucospilota_KAJ8046407.1
Holothuria_leucospilota_KAJ8046406.1
Holothuria_leucospilota_KAJ8046405.1
Holothuria_leucospilota_KAJ8046403.1
Holothuria_leucospilota_KAJ8047175.1
Lytechinus_variegatus_XP_041458460.1
Lytechinus_variegatus_XP_041458459.1
Lytechinus_variegatus_XP_041458461.1
Lytechinus_variegatus_XP_041458465.1
Holothuria_leucospilota_KAJ8047173.1
Lytechinus_variegatus_XP_041458467.1
Holothuria_leucospilota_KAJ8047170.1
Asterias_rubens_XP_033647727.1
Anneissia_japonica_XP_033106593.1
Lytechinus_variegatus_XP_041458464.1
Holothuria_leucospilota_KAJ8047172.1
Lytechinus_variegatus_XP_041458466.1
Asterias_rubens_XP_033648007.1
Pteria_penguin_mRNA.26954.1
Pteria_penguin_mRNA.26541.1
Pteria_penguin_mRNA.24497.1
Pteria_penguin_mRNA.23456.1
Pteria_penguin_mRNA.31126.2
Pteria_penguin_mRNA.30322.1
Pteria_penguin_mRNA.29669.1
Pteria_penguin_mRNA.31380.2
Pteria_penguin_mRNA.36661.1
Pteria_penguin_mRNA.34835.2
Owenia_fusiformis_CAH1795683.1
Owenia_fusiformis_CAH1795681.1
Owenia_fusiformis_CAH1788246.1
Owenia_fusiformis_CAH1775525.1
Hydra_vulgaris_XP_002165494.1
Nematostella_vectensis_XP_032236357.2
Pteria_penguin_mRNA.35085.1
Pteria_penguin_mRNA.34651.1
Pteria_penguin_mRNA.5231.2
Pteria_penguin_mRNA.35595.1
Pteria_penguin_mRNA.8835.2
Holothuria_leucospilota_KAJ8047168.1
Octopus_sinensis_XP_029643060.1
Rhopilema_esculentum_mRNA.RE07935
Asterias_rubens_XP_033646785.1
Pteria_penguin_mRNA.35629.1
Lytechinus_variegatus_XP_041458472.1
Anneissia_japonica_XP_033106552.1
Asterias_rubens_XP_033646517.1
Trichoplax_sp_RDD44554.1
Trichoplax_adhaerens_XP_002113109.1
100
200
300
400
500
Sequence length (aa)
UFboot
20
40
60
80
100
Motif ID
motif1
motif2
motif3
motif4
motif5
motif6
motif7
motif8
motif9
motif10
signal peptide
NTR domain

Figure S8
